# A membrane-associated phosphoswitch in Rad controls adrenergic regulation of cardiac calcium channels

**DOI:** 10.1172/JCI176943

**Published:** 2024-01-16

**Authors:** Arianne Papa, Pedro J. del Rivero Morfin, Bi-Xing Chen, Lin Yang, Alexander N. Katchman, Sergey I. Zakharov, Guoxia Liu, Michael S. Bohnen, Vivian Zheng, Moshe Katz, Suraj Subramaniam, Joel A. Hirsch, Sharon Weiss, Nathan Dascal, Arthur Karlin, Geoffrey S. Pitt, Henry M. Colecraft, Manu Ben-Johny, Steven O. Marx

**Affiliations:** 1 Division of Cardiology, Department of Medicine, and; 2 Department of Physiology and Cellular Biophysics, Vagelos College of Physicians and Surgeons, Columbia University, New York, New York, USA.; 3 Faculty of Medicine and; 4 Faculty of Life Sciences, Tel Aviv University, Tel Aviv, Israel.; 5 Department of Biochemistry and Molecular Biophysics, Vagelos College of Physicians and Surgeons, Columbia University, New York, New York, USA.; 6 Cardiovascular Research Institute and Department of Medicine, Weill Cornell Medical College, New York, New York, USA.; 7 Department of Pharmacology and Molecular Signaling, Vagelos College of Physicians and Surgeons, Columbia University, New York, New York, USA.

**Keywords:** Cardiology, Calcium channels, Cardiovascular disease, Excitation contraction coupling

## Abstract

The ability to fight or flee from a threat relies on an acute adrenergic surge that augments cardiac output, which is dependent on increased cardiac contractility and heart rate. This cardiac response depends on β-adrenergic–initiated reversal of the small RGK G protein Rad–mediated inhibition of voltage-gated calcium channels (Ca_V_) acting through the Ca_v_β subunit. Here, we investigate how Rad couples phosphorylation to augmented Ca^2+^ influx and increased cardiac contraction. We show that reversal required phosphorylation of Ser^272^ and Ser^300^ within Rad’s polybasic, hydrophobic C-terminal domain (CTD). Phosphorylation of Ser^25^ and Ser^38^ in Rad’s N-terminal domain (NTD) alone was ineffective. Phosphorylation of Ser^272^ and Ser^300^ or the addition of 4 Asp residues to the CTD reduced Rad’s association with the negatively charged, cytoplasmic plasmalemmal surface and with Ca_V_β, even in the absence of Ca_V_α, measured here by FRET. Addition of a posttranslationally prenylated CAAX motif to Rad’s C-terminus, which constitutively tethers Rad to the membrane, prevented the physiological and biochemical effects of both phosphorylation and Asp substitution. Thus, dissociation of Rad from the sarcolemma, and consequently from Ca_V_β, is sufficient for sympathetic upregulation of Ca^2+^ currents.

## Abstract 2

An electrostatic mechanism that switches between membrane-associated and membrane-dissociated Rad regulates adrenergic stimulation of calcium channels.

## Graphical abstract

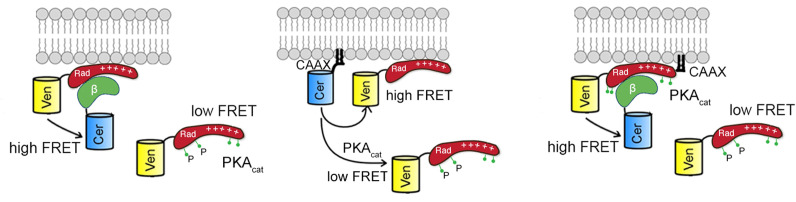

## Introduction

Calcium influx through voltage-gated L-type Ca^2+^ channels (Ca_v_1.2) is an essential link in the chain of signals that trigger the contraction of every heartbeat. Norepinephrine released from sympathetic neurons innervating the heart and circulating epinephrine released from the adrenal glands bind to cardiac β-adrenergic receptors, initiating an intracellular signaling cascade that generates cAMP and activates protein kinase A (PKA). Activated PKA phosphorylates various targets that control Ca^2+^ influx and intracellular Ca^2+^ concentration (1). The PKA-induced increase in Ca^2+^ current is obligatory for the positive inotropic cardiac response to β-adrenergic receptor agonists (2).

The mechanism underlying adrenergic regulation of Ca^2+^ channels had been elusive despite being investigated for decades (3–5). The consensus mechanism postulated that the regulation involved direct phosphorylation of Ca_V_1.2 pore-forming α_1_ or auxiliary β subunits on specific Ser and/or Thr residues. We showed, however, that phosphorylation of neither the α_1_ subunit nor the β subunit is required (6–10). Rather, the critical PKA target is Rad (2, 6), a small G protein (11) that basally inhibits a subpopulation of cardiac Ca_V_1.2 channels through an interaction with the auxiliary β subunit (12, 13). Knockout of Rad causes an increase in Ca^2+^ currents in cardiomyocytes, phenocopying the increase in Ca^2+^ currents upon adrenergic stimulation (14, 15). PKA phosphorylates Rad (16) on 4 Ser residues, 2 Ser in the N-terminus and 2 Ser in the C-terminus (6), and this releases Rad from Ca_V_β, disinhibiting the associated, channel-forming Ca_V_1.2 α subunit. Substitution of these 4 Ser residues in Rad with nonphosphorylatable Ala residues prevents adrenergic regulation of Ca_V_1.2, both when the subunits are expressed heterologously (6, 17–19) and endogenously in the heart (2).

The precise molecular mechanism by which Rad phosphorylation results in its release from Ca_V_β subunits remains undetermined. Ca_V_β binding to α_1C_, which is obligatory for PKA activation of Ca_V_1.2 (7), stabilizes an open-probability-gating (*P_O_*-gating) mode by a mechanism that requires a rigid linker between the β subunit binding site in the Ca_V_1.2 α subunit I-II loop and the preceding domain I channel pore (17). Although β_2_ predominates (20–25), cardiomyocytes express β_2_ and β_3_ Ca_V_β isoforms. These otherwise homologous β subunit isoforms differ substantially at their N- and C-termini and in their HOOK region between the conserved guanylate kinase (GK) and Src-homology 3 (SH3) domains (25), so β-adrenergic upregulation of Ca^2+^ currents might vary, depending on the specific β subunit. Favoring that possibility, the augmentation of Ca^2+^ influx by β-adrenergic agonists is more robust in β_2_-dominated cardiomyocytes than in tissues where other β subtypes predominate (26). Additionally, it was reported that adrenergic stimulation of Ca^2+^ channels was attenuated in β_2_-null cardiomyocytes, but not β_3_-null cardiomyocytes, suggesting that there is an exclusive role for the β_2_ subunit in the modulation of channel currents by β-adrenoceptor stimulation (22). Here, by knocking out the endogenous cardiomyocyte β_2_ subunit and expressing other Ca_V_β subunit isoforms in its absence, we show that the extent of adrenergic regulation of Ca^2+^ channels is independent of the specific Ca_V_β isoform.

Other regulatory details have also been elusive. It was postulated that Rad simultaneously binds to the sarcolemma and the Ca_V_β subunit (27–31). Here, we show that the effects of the phosphorylation of Ser and the mutation of Ser to Asp in the Rad C-terminal domain (CTD) are explained by the change in charge within the Rad CTD, strongly reducing the association of the Rad CTD with the cytoplasmic surface of the sarcolemma. The addition to the CTD of the CAAX motif that gets prenylated tethers Rad to the membrane (28) and prevents Rad’s displacement by phosphorylation; it also completely blocks adrenergic stimulation of Ca^2+^ currents. Thus, adrenergic stimulation of Ca^2+^ channels is based on an electrostatic mechanism that switches between membrane-associated and membrane-dissociated Rad. Similar signaling mechanisms based on electrostatic interactions with the negatively charged cytoplasmic face of the plasma membrane and specifically with negatively charged phosphoinositides have been proposed in the regulation of cell growth and differentiation (32–34).

## Results

**Figure 1:**
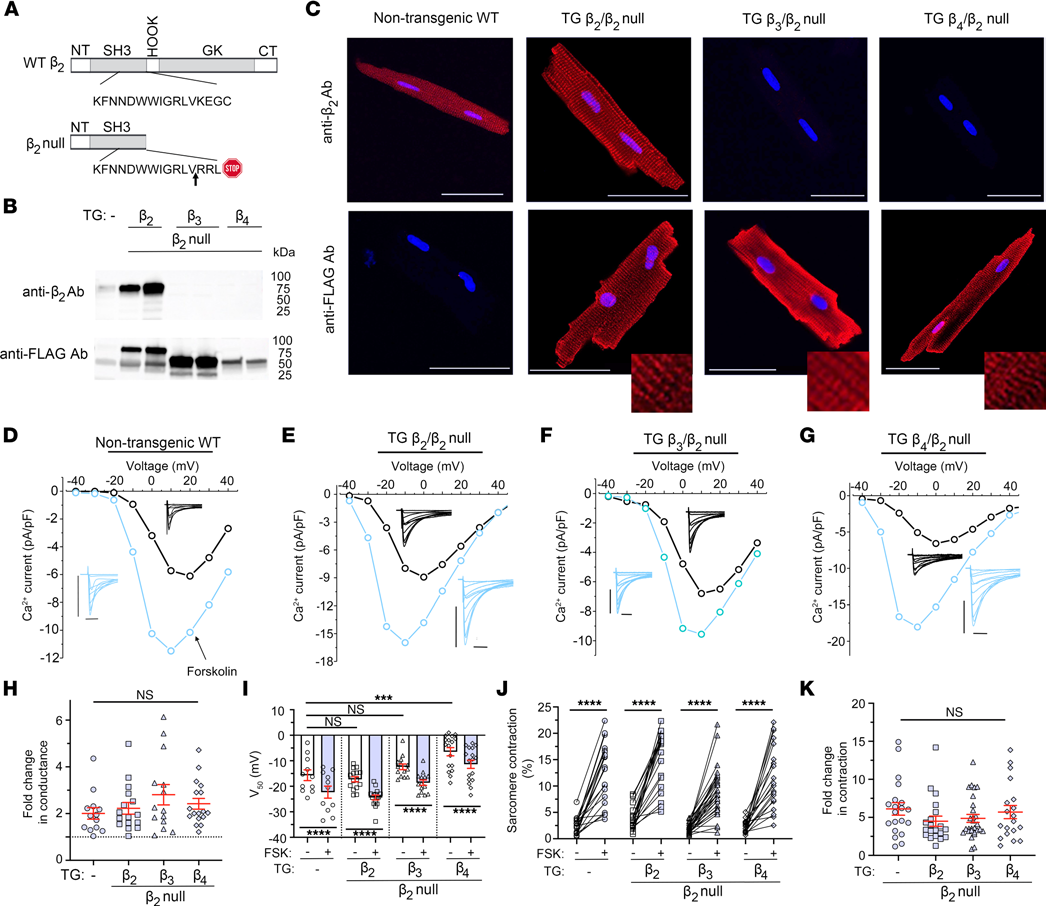
Extent of adrenergic regulation of Ca^2+^ channels is independent of a specific of Ca_V_β subunit isoform. (**A**) Schematic depicting Ca_V_β domains. NT, N-terminus; CT, C-terminus; Src homology 3 (SH3) domain, a conserved guanylate kinase (GK) domain, and a variable and flexible HOOK region that connects them. In β_2_-null mice, a frame-shift insertion causes an early termination at the end of the SH3 domain. (**B**) Anti-β_2_ and anti-FLAG immunoblots. (**C**) Anti-FLAG and anti-β_2_ immunofluorescence of nontransgenic WT and transgenic (TG) FLAG-β–expressing cardiomyocytes. Nuclear staining with DAPI. Scale bars: 50 μm. Representative of 3 similar experiments. Insets, ×4 enlargement to show striated pattern of expression. (**D**–**G**) Exemplar current-voltage relationships of Ca^2+^ channels in the absence (black trace) and presence of forskolin (blue trace). Insets: Exemplar whole-cell Ca_V_1.2 currents. Pulses from –60 mV to +10 mV before (black traces) and 3 minutes after (blue traces) forskolin. Horizontal scale bars = 50 ms, vertical scale bars = 10 pA/pF. (**H**) Fold change at –20 mV in peak current caused by forskolin (FSK). Mean ± SEM. *P* = not significant (NS) by 1-way ANOVA; *n* = 13, 16, 14, and 17 cardiomyocytes from 7, 3, 4, and 6 mice. TG, transgenic. (**I**) Boltzmann function parameter, *V_50_*, before and after FSK. Mean ± SEM. Statistical analysis among non-TG, β_2_, β_3_, and β_4_: *P* < 0.0001 by 1-way ANOVA; ***P < 0.001 by Šidák’s multiple-comparison test. Statistical analysis of no FSK versus FSK: ****P < 0.0001 by paired, 2-tailed *t* test. (**J**) Field stimulation–induced change in sarcomere contraction. ****P < 0.0001 by paired, 2-tailed *t* test. (**K**) Forskolin-induced fold change in sarcomere length. Mean ± SEM. *n* = 20, 20, 28, and 19 from 3, 3, 3, and 3 mice, from left to right. *P* = not significant by 1-way ANOVA.

Calcium channel β_2_ subunit–knockout (*Cacnb2*-knockout) mice were created using CRISPR by inserting a frameshift mutation causing early termination after the SH3 domain of the β_2_ protein within the indispensable exon 5 of the endogenous *Cacnb2* locus (Figure 1A). The homozygous constitutive global β_2_-null mice were not viable, as was previously reported (35). We therefore created transgenic mouse lines with cardiac-specific constitutive expression of FLAG-epitope tagged β_2B_, β_3_, and β_4_ subunits that were crossed with heterozygous β_2_-null mice to create heterozygous β_2_-null/transgenic FLAG-β_2B_, FLAG-β_3_, or FLAG-β_4_ mice. Finally, these cross-bred mice were crossed with heterozygous β_2_-null mice to achieve the endogenous β_2_-null background with cardiac-specific transgenic expression of either FLAG-tagged β_2B_, β_3_, or β_4_ (Figure 1B). Constitutive transgenic cardiac-specific expression of β_2B_, β_3_, or β_4_ subunits completely rescued the embryonic lethality of the homozygous global loss of the endogenous β_2_ subunits.

We assessed the impact of different β subunit isoforms on Ca_V_1.2 subcellular localization and functional expression in cardiomyocytes using 3 complementary approaches. First, immunofluorescence experiments with anti-FLAG antibody on fixed cardiomyocytes indicated that all β subunits displayed a similar striated pattern consistent with surface membrane distribution and localization in transverse tubules (Figure 1C). Second, we tested the electrophysiological properties of Ca^2+^ channels in ventricular cardiomyocytes isolated from wild-type (WT) mice and mice expressing either FLAG-β_2B_, FLAG-β_3_, or FLAG-β_4_ subunits in the homozygous β_2_-null background. In β_2B_, β_3_, or β_4_ subunit–expressing transgenic ventricular cardiomyocytes, the adenylyl cyclase activator forskolin increased the amplitude of the Ca^2+^ currents and shifted the *V_50_* for Ca^2+^ channel activation in a hyperpolarizing direction, similarly to the effects of forskolin in WT ventricular cardiomyocytes (Figure 1, D–I). Basal electrophysiological properties, including conductance density, were similar among the 4 groups (Supplemental Figure 1A; supplemental material available online with this article; https://doi.org/10.1172/JCI176943DS1), although the *V_50_* for activation of Ca^2+^ channels incorporating the β_4_ subunit, an exception, was shifted to a more depolarized voltage (Figure 1I). Third, we measured changes in pacing-induced sarcomere length before and after exposure to forskolin. The field-stimulated basal and forskolin-augmented contractions of cardiomyocytes isolated from mice expressing WT or transgenic β_2B_, β_3_, or β_4_ subunits were not significantly different (Figure 1, J and K). In cardiomyocytes, there is an inverse relationship between total peak current and isoproterenol-induced or forskolin-induced fold increases in Ca^2+^ currents (7, 36). In cardiomyocytes isolated from nontransgenic mice and transgenic mice with β_2B_, β_3_, or β_4_ subunit–expressing ventricular cardiomyocytes, we observed an inverse relationship between basal conductance density and forskolin-induced increases in Ca^2+^ conductance (Supplemental Figure 1B). We conclude that β_2_, β_3_, and β_4_ subunits are all capable of mediating the PKA-dependent augmentation of Ca^2+^ currents in the heart. Thus, the critical components within Ca_V_β subunits that regulate Rad-mediated adrenergic increase in Ca_V_1.2 currents reside within the homologous regions in all β subunits and likely exclude the N- and C-termini and their HOOK regions that harbor the Ca_V_β isoform–specific differences.

### Essential contribution of Rad CTD phosphorylation but not NTD phosphorylation for Ca_V_1.2 regulation.

**Figure 2:**
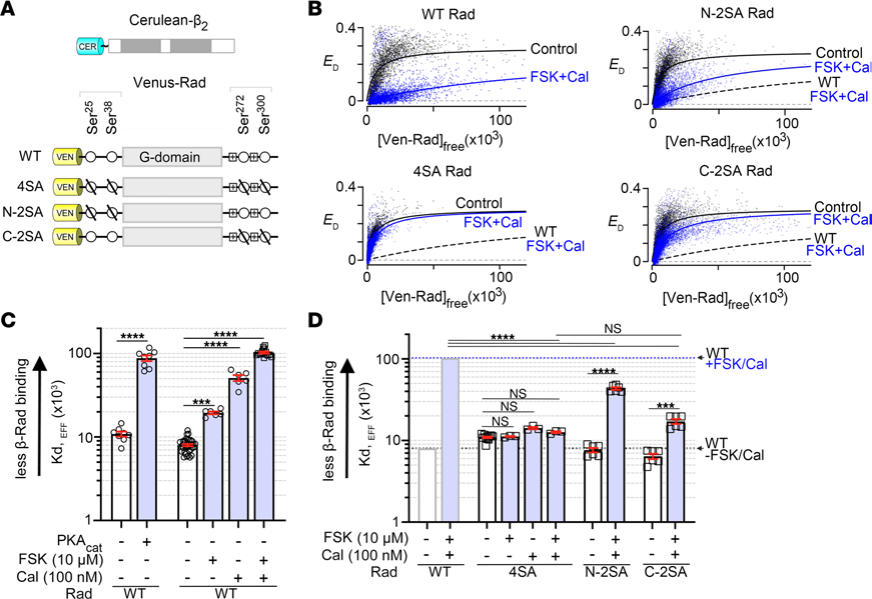
Effects of phosphorylation of Venus-Rad on its binding to Cerulean-β_2B_, as monitored by FRET. (**A**) Schematics of the FRET pairs, Cerulean (Cer)-β_2B_ with Venus (Ven)-WT Rad, and Ven-4SA, Ven-N-2SA, and Ven-C-2SA Rad. Small circles are phosphorylation sites. 4SA: Ala substitutions of Ser^25^, Ser^38^, Ser^272^, and Ser^300^; N-2SA: Ala substitutions of Ser^25^ and Ser^38^; C-2SA: Ala substitutions of Ser^272^ and Ser^300^. (**B**) FRET efficiency (*E_D_*) between Ven-conjugated WT or Ven-conjugated mutant Rad and Cer-β_2B_ is plotted against the free concentration of Ven-Rad, in the absence (control, black) and the presence (blue) of 10 μM forskolin (FSK) and 100 nM calyculin A (Cal). The 1:1 binding isotherms are fit to the data (solid lines). The dashed lines are fits to the data from FSK/Cal–treated WT Rad cells. (**C** and **D**) Graphs summarizing mean *K_d,EFF_* for the binding of β_2B_ and WT Rad, 4SA Rad, N-2SA Rad, and C-2SA Rad, in absence and presence of PKA_cat_, FSK, and Cal. Error bars are SEM. The significance of the differences versus no treatment are *P* < 0.0001 by 1-way ANOVA; ***P < 0.001, ****P < 0.0001 by Šidák’s multiple-comparison test. For **C**, *n* = 8, 8, 36, 6, 6, and 14 from left to right. For **D**, WT data are same as **C**, starting at 4SA Rad: *n* = 12, 3, 3, 3, 6, 6, 6, and 6 from left to right.

We used a flow cytometric, Förster resonance energy–transfer (FRET) assay to measure binding between Cerulean-tagged β_2B_ subunits and Venus-tagged WT Rad expressed in HEK293 cells (Figure 2, A and B). Cerulean and Venus are FRET partners. As previously shown, expression of the PKA catalytic (PKA_cat_) subunit markedly weakened this interaction, as measured by FRET efficiency, *E_D_* (Figure 2B) and relative dissociation constant (*K_d,EFF_*) (6). To acutely activate PKA regulation in HEK293 cells, we used forskolin as we did in cardiomyocytes. We found that forskolin (10 μM or 50 μM) reduced the Ca_V_β-Rad interaction compared with DMSO vehicle controls, although the effect was substantially less compared with overexpression of the PKA_cat_ subunit (Figure 2C and Supplemental Figure 2, A and B). Calyculin A, an inhibitor of protein phosphatase 1 and 2, reduced the binding of Ca_V_β and Rad more than forskolin, and the combination of calyculin A and forskolin reduced the binding even further, an effect similar to that of PKA_cat_ subunit overexpression (Figure 2, B and C, and Supplemental Figure 2B). The combination of forskolin and calyculin A had no effect on the binding of Cerulean-tagged Ca_V_β and Venus-tagged 4SA-Rad, in which the 4 PKA phosphorylation sites, Ser^25^ and Ser^38^ in the N-terminal domain (NTD) and Ser^272^ and Ser^300^ in the CTD, are replaced with Ala (Figure 2, A, B, D, and Supplemental Figure 2B).

We compared the roles of the NTD and CTD phosphorylation sites of Rad on mutants in which the Ser residues were eliminated from either domain. In HEK293 cells expressing fluorophore-tagged β_2B_ and C-2SA Rad with Ser^272^ and Ser^300^ mutated to Ala (Figure 2A), forskolin plus calyculin A reduced binding far less than in WT (Figure 2, B and D, and Supplemental Figure 2B). By contrast, in cells expressing fluorophore-tagged β_2B_ and N-2SA Rad with Ser^25^ and Ser^38^ mutated to Ala (Figure 2A), forskolin and calyculin reduced binding of Rad and Ca_V_β nearly as much as in WT (Figure 2, B and D, and Supplemental Figure 2B). Thus, phosphorylation-dependent dissociation requires Ser^272^ and Ser^300^ in the CTD. Nonetheless, phosphorylation of the NTD Ser makes a small contribution to the dissociation of Rad from β in HEK293 cells.

**Figure 3:**
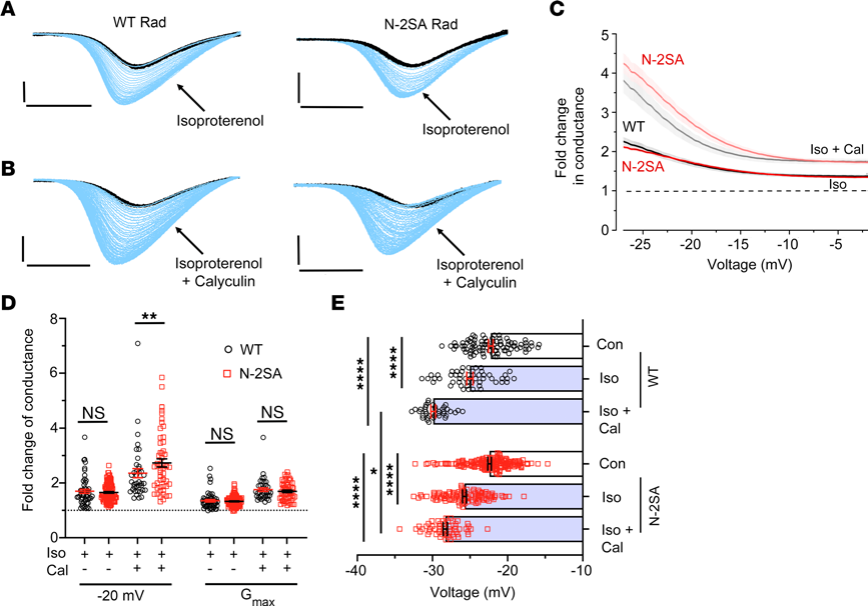
Effects in ventricular cardiomyocytes of removing sites for PKA phosphorylation from the Rad NTD on β-adrenergic agonist augmentation of Ca_V_1.2 currents. (**A** and **B**) Ba^2+^ currents elicited by voltage ramp every 3 seconds in WT and N-2SA Rad ventricular myocytes, with black traces obtained before and blue traces obtained after isoproterenol or isoproterenol plus calyculin A. Scale bars: 3 pA/pF (vertical) and 50 ms (horizontal). (**C**) Graph of fold change in Ba^2+^ conductance after isoproterenol (Iso) or isoproterenol plus calyculin (Iso + Cal) to Ba^2+^ conductance before treatment versus voltage of cardiomyocytes isolated from WT and N-2SA Rad mice. Mean ± SEM. For isoproterenol: WT: 51 cells, 7 mice, N-2SA: 112 cells, 11 mice; for calyculin: WT: 38 cells, 3 mice, N-2SA: 43 cells, 5 mice. (**D**) Fold change in peak current at –20 mV and *G_max_*. Mean ± SEM. Same sample size as in **C**. *P* < 0.0001 by 1-way ANOVA; **P < 0.001 by Šidák’s multiple-comparison test. (**E**) Boltzmann’s function parameter, *V_50_*, before and after isoproterenol, or isoproterenol and calyculin A. *P* < 0.0001 by 1-way ANOVA; *P < 0.05, ****P < 0.0001 by Šidák’s multiple-comparison test.

To explore this question in cardiomyocytes, we created N-2SA Rad–knockin mice in which the Ser^25^ and Ser^38^ of endogenous Rad were replaced by Ala residues (Supplemental Figure 3A). The expression in cardiomyocytes of mutant Rad and endogenous Ca_V_1.2 α_1c_ subunits was unaffected by the substitutions (Supplemental Figure 3B). We tested the electrophysiological properties of Ca^2+^ channels in ventricular cardiomyocytes isolated from WT and homozygous N-2SA Rad–knockin mice using a voltage-ramp protocol applied every 3 seconds with Ba^2+^ as the charge carrier (Figure 3, A and B). The ramp protocol enables the monitoring of agonist effects over time. Isoproterenol increased the Ca_V_1.2 currents of WT and N-2SA Rad ventricular cardiomyocytes and shifted the *V_50_* for activation toward hyperpolarized potentials equivalently (Figure 3, C–E); i.e., the 2 Ser susceptible to phosphorylation by PKA in the Rad NTD play at most a minor role in adrenergic regulation of Ca_V_.

A critical determinant of the magnitude of channel modulation is the balance between the competing activities of PKA and phosphatases. We exposed cells to a combination of isoproterenol and calyculin A. In both WT and N-2SA ventricular cardiomyocytes, the combination of isoproterenol and calyculin A produced substantially more enhancement of current than isoproterenol alone (Figure 3, C–E). The *V_50_* for activation was also shifted to a greater extent by combined isoproterenol and calyculin compared with isoproterenol alone. Once again, we conclude that phosphorylation of the 2 NTD Ser residues plays at most a minor role in the acute adrenergic enhancement of Ca_V_1.2 currents in ventricular cardiomyocytes.

### Effect of lowering the charge of Rad by substituting Asp for Ser on its binding to β.

**Figure 4:**
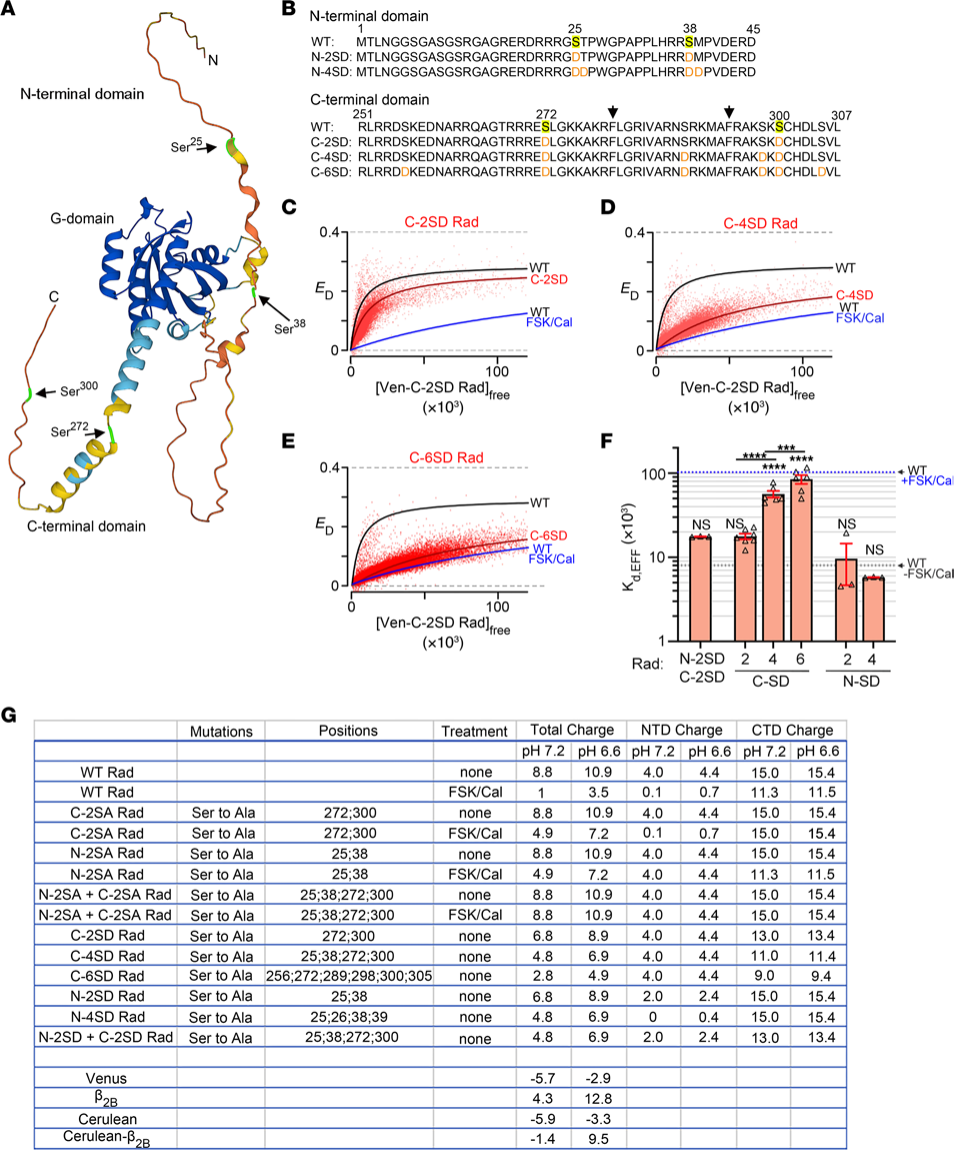
Effects on Venus-Rad binding to Cerulean-β_2B_ by the insertion of negatively charged Asp residues in the N- and C-termini of Rad. (**A**) Protein structure prediction with AlphaFold of human Rad (P55042) (60, 61). (**B**) Protein sequences of N-terminus and C-terminus of Rad, showing phosphorylated residues (highlighted yellow) and residues substituted with Asp (red font). Arrowheads mark hydrophobic residues in the basic-hydrophobic motif of Rad. (**C**–**E**) FRET efficiency (*E_D_*) between Ven-Rad mutants and Cer-β_2B_ is plotted against the free concentration of Ven-C-2SD, C-4SD, and C-6SD Rad. The red line fits a 1:1 binding isotherm for C-2SD, C-4SD, and C-6SD Rad. The black and blue lines are the 1:1 binding isotherm for WT-Rad in the absence and presence of FSK + Cal (same as Figure 2B). (**F**) Graph summarizing mean *K_d,EFF_* for the binding of β_2B_ and WT and mutant Rad. Error bars are SEM. Black and blue dashed lines are mean values of WT Rad without and with FSK + Cal (from Figure 2). *P* < 0.0001 by 1-way ANOVA; ****P < 0.0001 compared with WT Rad without FSK + Cal by Dunnett’s multiple-comparison test. *n* = 3, 7, 6, 6, 3, and 3 from left to right. (**G**) Table showing changes in charge induced by either treatment with forskolin (FSK) and calyculin (Cal) or substitution of Asp residues in full-length Rad, in the N-terminal domain of Rad (NTD, residues 1–45), or in the C-terminal domain of Rad (CTD, residues 251–307), calculated using https://protcalc.sourceforge.net The change in charge on phosphorylated Ser residues by the addition of a phosphate group is –1.96 at pH 7.2, and –1.86 at pH 6.6, assuming pKa2 = 5.8 (62).

The region of Rad bound to Ca_V_β is putatively within the centrally located guanine nucleotide–binding domain (G-domain) of Rad (37, 38), not in the CTD, the region of functionally relevant phosphorylation (Figure 4A). The CTD is likely intrinsically disordered, which allows for large conformational changes that can support dynamic signaling (39). To test the effects of charge as opposed to a possible specific effect of phosphorylation in the CTD, we substituted Ser^272^ and Ser^300^ with aspartic acid (Asp) residues (C-2SD) (Figure 4B). These substitutions, which added only 2 negative charges compared with the 4 negative charges added by 2 phosphates, evoked only a small decrease in the interaction between Ca_V_β and Rad (Figure 4, B and C), leading us to speculate that the increase in net negative charges is critical. Therefore, to achieve a similar (and greater) net increase in negative charges compared with the addition of 2 phosphates, we substituted 2 more Ser with Asp (C-4SD) and 4 more Ser with Asp (C-6SD) within the CTD (Figure 4B). The additional Ser residues have not been identified as phosphorylation sites but provide an opportunity to substitute homologous side chains that confer an additional negative charge. We utilized a similar strategy within the N-terminus. The apparent dissociation constant of the complex increased with the number of substituted Asp in the C-terminus (Figure 4, C–F) and hence with decreased net positive charge of Rad. The substitution of 6 Asp for Ser (C-6SD) was nearly as effective in reducing binding as was the combination of forskolin and calyculin A, which induces phosphorylation of the 2 native CTD Ser^272^ and Ser^300^. By contrast, substituting Asp solely in the NTD, as in N-2SD and N-4SD, or in addition to 2 CTD Asp (combined N-2SD and C-2SD), had no significant effect on binding (Figure 4F), despite the lowered positive charge overall. These results are consistent with the minor role of phosphorylation of NTD Ser in the adrenergic regulation of Ca_V_1.2 currents. Instead, the local charge of the Rad CTD is a critical determinant for Rad association with Ca_V_β. Moreover, these findings highlight the difference between Asp substitution (change in charge of –1) compared with phosphorylation (change in charge of –2).

### Phosphorylation of Rad CTD reduces association of Rad with the plasma membrane.

Anionic phospholipids in the inner leaflet of the plasma membrane generate an electrostatic surface potential estimated to be approximately –35 mV (40). Assuming that surface potential and pH 7.2 in the bulk cytoplasm, the equilibrium pH at the membrane surface would be 6.6, which would increase the charge of proteins at the surface largely due to the charging of histidyl residues. If we take the CTD of Rad as the 57 residues from 251 to 307, the C-terminus contains 16 more Arg and Lys than Asp and Glu and no His (Figure 4, A and B). The estimated net charge of the CTD at pH 7.2 is 15 and at pH 6.6, 15.4 (Figure 4G). The CTD also includes 16 hydrophobic residues. This combination of basic and hydrophobic residues favors attachment to the plasmalemma (27, 28, 33, 41, 42). Candidate phospholipid bilayer–binding motifs can be identified using web-based prediction tools such as a basic-hydrophobic (BH) algorithm score (34, 43). CTD residues 270 to 297 have BH scores well above the threshold of 0.6 (Supplemental Figure 4A). The propensity of such positively charged motifs to attach to the plasmalemma is lowered by a reduction in their charge, commonly by phosphorylation (34). We determined whether in fact phosphorylation of Rad affected its association with the plasma membrane.

**Figure 5:**
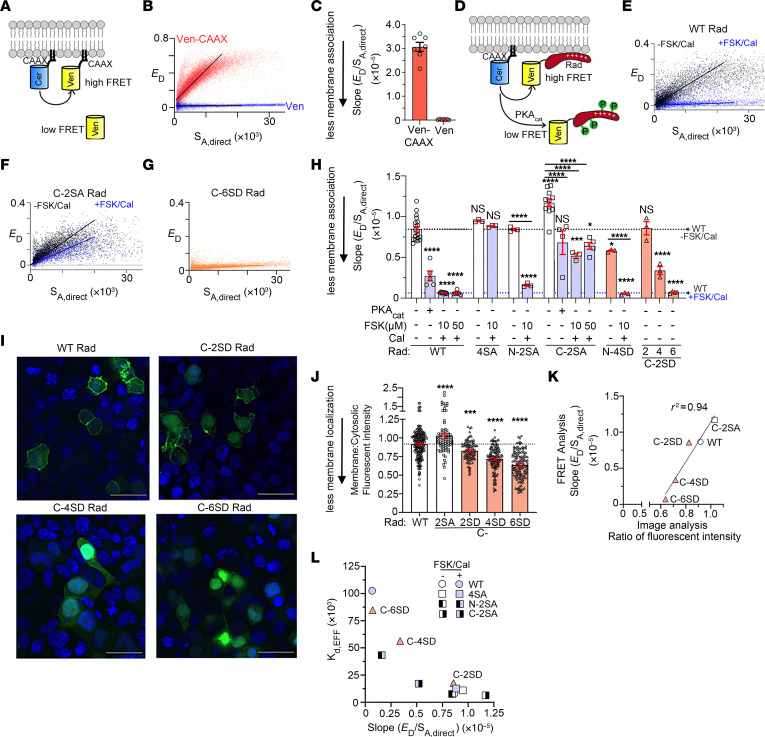
Effects on Venus-Rad binding to the membrane of phosphorylation and insertion of negatively charged Asp residues. (**A**) FRET biosensor for membrane binding. Cerulean and Venus fluorescent proteins were conjugated with CAAX. (**B**) FRET efficiency (*E_D_*) is plotted against *S_A,direct_*, the fluorescence intensity of the acceptor (Venus), directly excited. Lines are linear slope using least-squares fit. (**C**) Slope of *E_D_* between Cer-CAAX and Ven-CAAX or Ven alone. Mean ± SEM. ****P < 0.0001 by 2-tailed, unpaired *t* test. *n* = 7. (**D**) Shown are Cer-CAAX and WT Rad or mutant Rad conjugated to the Venus fluorescent protein. High FRET signal is detected when both proteins are colocalized at the membrane. (**E**) *E_D_* is plotted against *S*_A,direct_ of Ven-WT Rad, either untreated or treated with 10 μM forskolin plus 100 nM calyculin A. (**F**) As in **E**, with C-2SA Rad. (**G**) As in **E**, with C-6SD Rad. (**H**) FRET binding studies of Ven-conjugated proteins to membrane. Mean ± SEM. Statistics for comparison to control column (WT without PKA, FSK or Cal). *P* < 0.0001 by 1-way ANOVA; *P < 0.05, ***P < 0.001, ****P < 0.0001 by Dunnett’s test. *n* = 20, 5, 9, 6, 3, 3, 3, 3, 11, 4, 3, 5, 3, 3, and 4 from left to right. (**I**) Fluorescence of GFP-tagged WT and mutant Rad expressed in HEK293 cells. Nuclear staining with DAPI. Scale bars: 32 μm. (**J**) Ratio of membrane and cytosolic fluorescence intensities for WT and mutant Rad protein. Mean ± SEM. *P* < 0.0001 by 1-way ANOVA; ***P < 0.001, ****P < 0.0001 by Šidák’s test compared with WT Rad. *n* = 223, 84, 103, 135, and 143 cells from left to right. (**K**) Relationship between fluorescence image analysis and FRET analysis. Line was fit by linear regression. (**L**) Correlation between Rad membrane association and β-Rad binding without and with 10 μM forskolin and 100 nM calyculin for WT Rad and Rad mutants 4SA, N-2SA, C-2SA, C-2SD, C-4SD, and C-6SD.

To generate a FRET biosensor for detecting the membrane localization of Rad, we tethered the Cerulean fluorescent protein to the plasma membrane localization motif CAAX (Cerulean-CAAX) (Figure 5A). The CAAX motif has an invariant Cys, 2 aliphatic amino acids (A_1_ and A_2_), and one of several amino acids in the terminal X position (44). Proteins with C-terminal CAAX motifs undergo posttranslational prenylation modification through the addition of either farnesyl or geranylgeranyl isoprenoids to the Cys residues of the CAAX motif, thus promoting strong membrane association (45). To test whether tethered Cerulean-CAAX can detect membrane localization of a second protein, we also conjugated CAAX to the Venus fluorescent protein (Venus-CAAX). The FRET signal from Cerulean-CAAX to Venus-CAAX, expressed in HEK293 cells, was strong, while the FRET signal from Cerulean-CAAX to untethered Venus was weak (Figure 5, B and C).

Using this approach to detect colocalization of proteins at the membrane, we coexpressed Cerulean-CAAX and Venus-conjugated Rad (Figure 5D). At baseline, we detected robust FRET from Cerulean-CAAX to Venus-Rad (Figure 5, E and H). The fluorescence of directly excited Venus-Rad, *S_A,direct_*, is proportional to the total number of Venus-Rad molecules in the cell. The slope of the FRET efficiency, *E_D_*, versus *S_A,direct_* is itself proportional to the fraction of Venus-Rad associated with the membrane (see Supplemental Methods). Coexpression of the PKA_cat_ subunit or treatment of the cells with the combination of forskolin (10 μM or 50 μM) and calyculin A markedly reduced FRET and, hence, the association of Venus-Rad with the membrane (Figure 5, E and H, and Supplemental Figure 4, B and C). Ala substitutions of both NTD and CTD Ser residues (4SA-Rad), but not Ala substitutions of NTD Ser residues alone (N-2SA), prevented the reduction in membrane localization induced by forskolin and calyculin (Figure 5H). In comparison with WT Rad, Ala substitution of only the 2 CTD Ser residues, Ser^272^ and Ser^300^, enhanced the basal association of Rad with the plasma membrane (Figure 5H). The reason for this enhancement of membrane localization is not clear, considering that the 4SA-Rad mutant did not have a similar effect. The effects of PKA_cat_ expression or exposure to forskolin and calyculin were attenuated for C-2SA Rad (Figure 5, F and H, and Supplemental Figure 4, D and E). Replacing Ser^272^ and Ser^300^ with the Asp residues was not sufficient to reduce membrane localization of Rad, but the addition of more Asp residues to the CTD of Rad reduced localization of Rad to the plasma membrane in a graded fashion (Figure 5, G and H, and Supplemental Figure 4, F and G). In summary, phosphorylation of the Ser residues (net increase of 4 negative charges) or the substitution of 4 or more Asp for Ser (net increase of ≥4 net negative charges) in the CTD reduces membrane localization of Rad.

In a second approach to assess membrane localization, we expressed GFP-tagged WT Rad and GFP-tagged Asp-substituted Rad in HEK293 cells (Figure 5I). We quantified the radially averaged fluorescence intensity profile of GFP-Rad at the membrane and within the cytoplasm. The addition of negatively charged Asp residues to the CTD of Rad reduced the localization of Rad at the membrane (Figure 5J). The localization of the charge variants of Rad to the plasmalemma determined by fluorescence microscopy and that determined by FRET were highly correlated (Figure 5K).

We compared the relative dissociation constant for Rad binding to the Ca_V_β subunit with the amount of membrane association based on FRET between Venus-tagged Rad and Cerulean-CAAX (slope). Binding between Rad and β decreases with decreasing membrane localization (Figure 5L). Two possibilities for decreased binding of Rad to Ca_V_β are that phosphorylation and substitution of Asp in the CTD of Rad (a) might change the Rad-Ca_V_β binding interface or (b) might change the concentration of Rad close to the membrane, where in cardiomyocytes, Ca_V_β, bound to Ca_V_α, is also close to the membrane.

### Constitutive Rad plasma membrane localization prevents phosphorylation-dependent changes in β binding.

**Figure 6:**
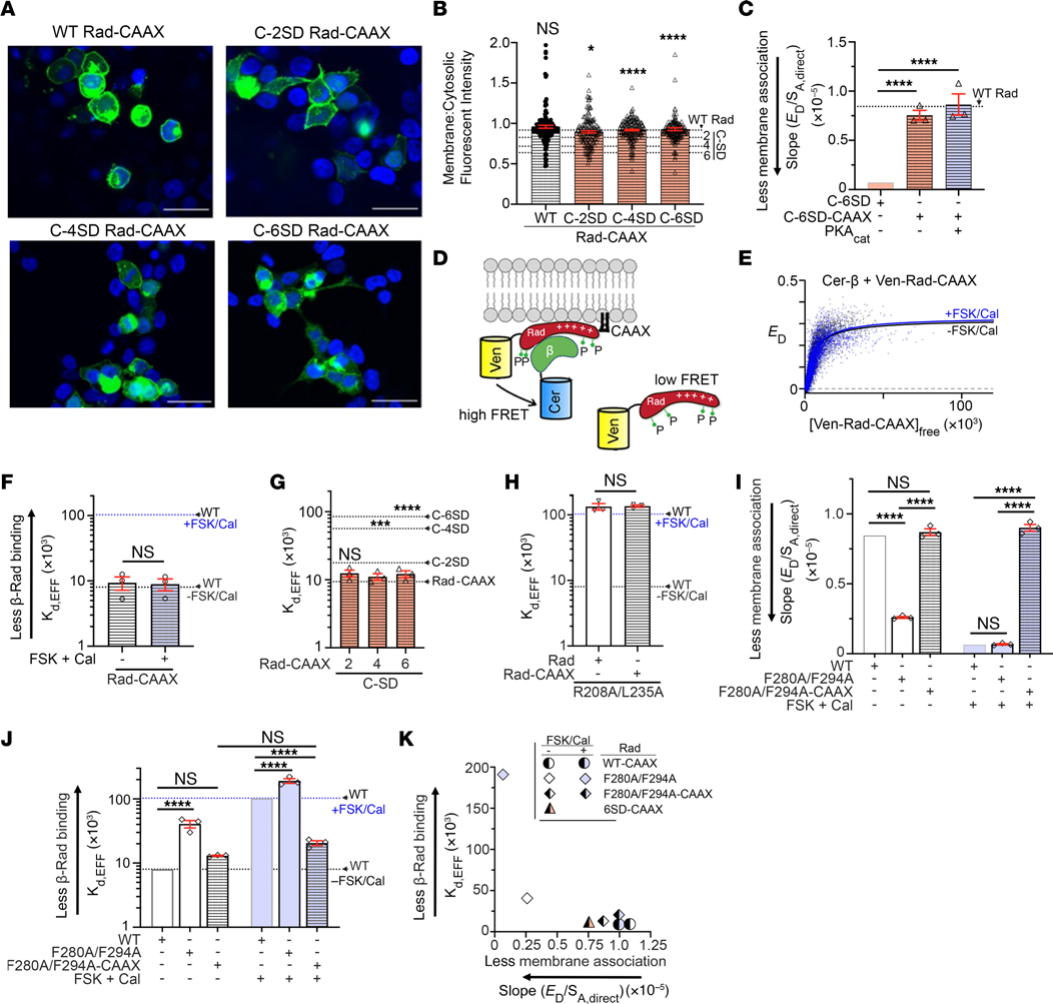
Effects of tethering of Rad to the plasma membrane via CAAX motif. (**A**) Fluorescence of GFP-tagged WT and mutant Rad-CAAX proteins. Nuclear staining with DAPI. Scale bars: 32 μm. (**B**) Ratio of membrane and cytosolic fluorescence intensities. Mean ± SEM. Dashed lines are mean values of WT Rad, and C-2SD, C-4SD, and C-6SD (from Figure 5). Statistical comparisons to non–CAAX-conjugated constructs. *P* < 0.0001 by 1-way ANOVA; *P < 0.05, ****P < 0.0001 by Šidák’s test. *n* = 183, 167, 180, and 175 cells from left to right. (**C**) FRET binding studies of Ven-conjugated C-6SD proteins to membrane. C-6SD bar is same as in Figure 5H. Dotted line is value for WT Rad (from Figure 5H). Mean ± SEM. *P* < 0.0001 by 1-way ANOVA; ****P < 0.0001 by Dunnett’s test. *n* = 4, 3, and 3 from left to right. (**D**) Schematic of Cer-β and Ven-Rad-CAAX. (**E**) FRET efficiency between Ven-Rad-CAAX and Cer-β is plotted against the total concentration of Ven-Rad-CAAX. (**F**) Mean *K_d,EFF_* for binding of Rad-CAAX to β. Error bars are SEM. Differences not significant by 2-tailed, unpaired *t* test. *n* = 3 and 3 from left to right. (**G**) Mean *K_d,EFF_* for binding of CAAX-conjugated mutant Rad proteins to β. Error bars are SEM. Statistical comparisons to non–CAAX-conjugated constructs. *P* < 0.0001 by 1-way ANOVA; ***P < 0.001, ****P < 0.0001 by Šidák’s test. (**H**) Mean *K_d,EFF_* for binding of β to R208A/L235A Rad or R208A/L235A-CAAX Rad. Differences are not significant by 2-tailed, unpaired *t* test. (**I**) As in **C**, with F280A/F294A. WT and WT + FSK/Cal are the same data as in Figure 5H. Error bars are SEM. *P* < 0.0001 by 1-way ANOVA; ****P < 0.0001 by Šidák’s test. *n* = 20, 3, 3, 9, 3, and 3 from left to right. (**J**) Mean *K_d,EFF_* for binding of β to F280A/F294A Rad or F280A/F294A-CAAX Rad. Error bars are SEM. *P* < 0.0001 by 1-way ANOVA; ****P < 0.0001 by Šidák’s test. *n* = 36, 3, 3, 14, 3, and 3. (**K**) Correlation between Rad membrane association and β-Rad binding.

The C-termini of many members of the Ras superfamily of proteins, in contrast to the RGK GTPases, contain a CAAX prenylation motif that confers membrane binding independent of the basic-hydrophobic motif (46). The addition of a CAAX motif to a C-terminally deleted Rem1 that cannot bind to the membrane was previously shown to restore membrane localization (28). We tested whether the addition of the CAAX motif to Rad enables membrane binding even in the presence of added negative charges in the C-terminus. Both fluorescence imaging (Figure 6, A and B) and FRET (Figure 6C) analyses showed that the introduction of the CAAX motif completely overcomes the effects of either phosphorylation or the addition of Asp residues in the CTD on membrane localization of Rad (Figure 6, D–G).

Notwithstanding the importance of membrane localization of Rad, substituting Rad residues Arg^208^ and Leu^235^ with Ala prevents Rad binding to Ca_V_β (30, 37), which we confirmed using the FRET assay for detection of Rad and Ca_V_β interactions (Figure 6H). The addition of CAAX to the C-terminus of the R208A/L235A Rad mutant, moreover, failed to restore the binding of Rad and β subunits (Figure 6I). Thus, the constitutive tethering of Rad to the membrane could not restore the interaction between Rad and Ca_V_β perturbed by mutations at the Rad-β binding interface.

The presence of hydrophobic amino acid side chains together with positively charged Lys and Arg residues strengthen association with the plasma membrane and prevent a polybasic cluster from functioning as a nuclear localization sequence (33). Mutation of the hydrophobic residues Phe^280^ and Phe^294^ (arrows, Figure 4B) to Ala in the C-terminus of Rad reduced both membrane localization (Figure 6I) and Rad-β subunit interaction (Figure 6J). The addition of CAAX to this mutant Rad restored both basal membrane binding (Figure 6I) and Rad-β binding (Figure 6J). Because in this case Rad is constitutively tethered to the membrane via posttranslational modification of the CAAX motif, the forskolin/calyculin–induced changes in membrane localization (Figure 6I) and Rad-β interaction (Figure 6J) are nearly eliminated. As before, we compared the relative dissociation constants (*K_d,EFF_*) for various Rad-CAAX mutants binding to the Ca_V_β subunit with the amount of membrane association based on FRET (Figure 6K). Appending the CAAX motif to the C-terminus of both WT and mutant Rad restrained them in a membrane-bound and Ca_V_β-bound conformation. The finding that the binding of β_2B_ and Rad-CAAX is unaffected by forskolin and calyculin implies that phosphorylation or substitution of Asp in the CTD of Rad does not directly change the Rad-β binding interface.

### Disinhibition of Ca^2+^ channels requires a decrease in the membrane association of Rad.

We tested whether a change in membrane association is essential for adrenergic stimulation of Ca^2+^ channels. Consistent with the physiological function of Rad as an inhibitor of Ca^2+^ channels, coexpression in HEK293 cells of WT Rad with α_1C_ and β_2B_ decreased the amplitude of tail currents (Supplemental Figure 5, A and B). Furthermore, consistent with the findings above that Rad with Asp substitutions in the CTD binds less well to β, we observed that the amplitude of Ca_V_ tail currents increased as negative charges were added to the Rad CTD (Supplemental Figure 5, A and B).

**Figure 7:**
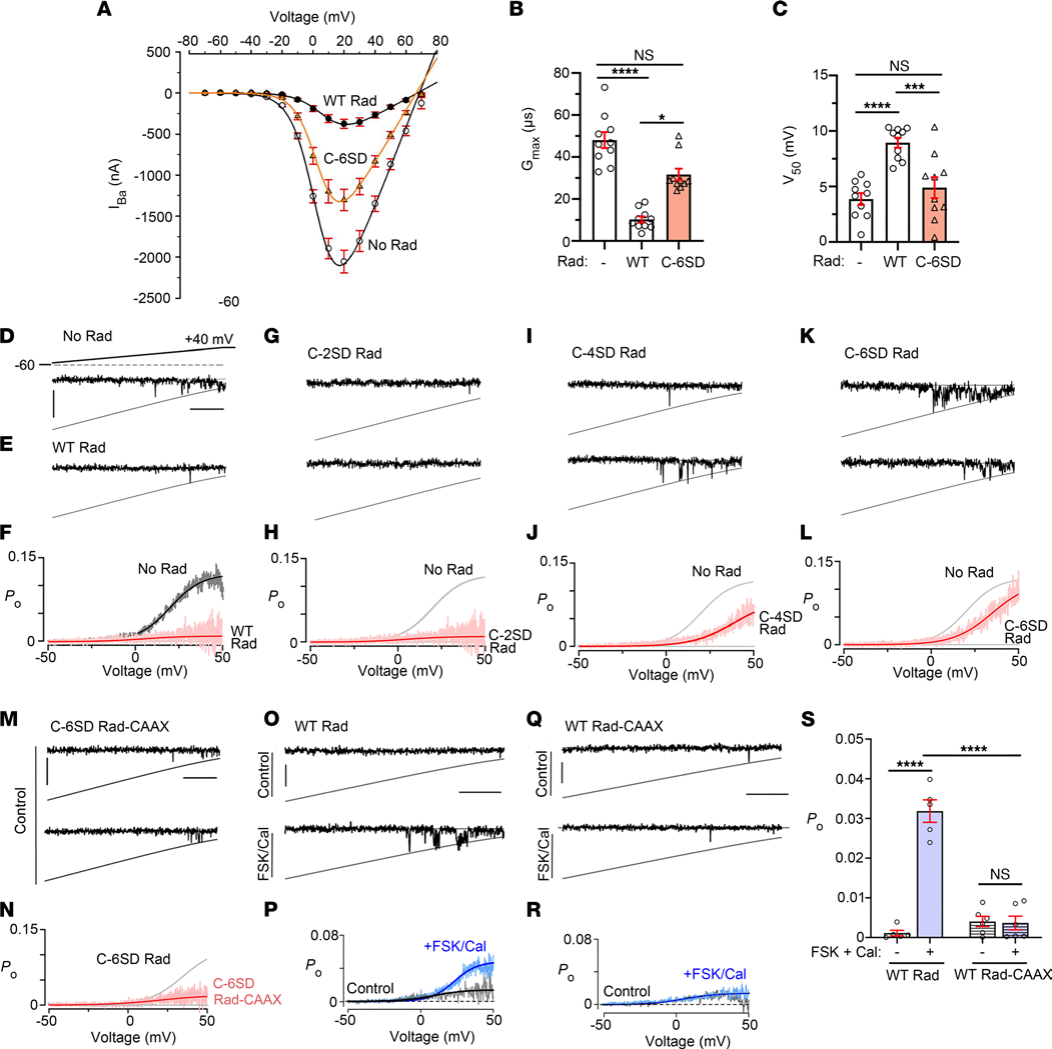
Effects of insertion of negatively charged Asp residues or CAAX in C-terminus of Rad on electrophysiological properties of Ca_V_1.2 channels. (**A**) Current-voltage (I-V) curves of Ba^2+^ currents (I_Ba_) in oocytes expressing α_1C_, β_2B_, α_2_δ, and no Rad, WT Rad, or C-6SD Rad. Net I_Ba_ values (mean ± SEM), obtained after subtraction of current in the presence of Cd^2+^, are shown. The solid lines are I-V curves drawn with the Boltzmann equation (see Supplemental Table 1). (**B**) Graph of whole-cell maximal Ba^2+^ conductance, *G_max_*. Bars show mean ± SEM. *n* = 10 oocytes in each group. *P* < 0.0001 by Kruskal-Wallis test; *P < 0.05, ****P < 0.0001 by Dunn’s multiple-comparison test. (**C**) Graph of *V_50_*, *P* < 0.0001 by 1-way ANOVA; ***P < 0.001, ****P < 0.0001 by Tukey’s multiple-comparison test. NS, *P* > 0.05. (**D**, **E**, **G**, **I**, and **K**) Single-channel Ba^2+^ currents are shown. Openings are downward deflections to the open level (slanted gray curves). Horizontal bar = 25 ms; vertical bar = 1 pA. (**F**, **H**, **J**, and **L**) Mean *P_o_* versus voltage relationship. *n* = 5 for all groups. Black and red lines are Boltzmann fits. (**M**) Single-channel Ba^2+^ currents for Ca_V_1.2 channels coexpressed with C-6SD-CAAX Rad. (**N**) Mean *P_O_* versus voltage relationship. *n* = 5 cells. Solid lines are Boltzmann fits. Horizontal bar = 25 ms; vertical bar = 1 pA. (**O** and **Q**) Single-channel Ba^2+^ currents are shown for Ca_V_1.2 channels coexpressed with WT Rad or WT Rad-CAAX in the absence or presence of FSK and Cal preincubated for 5 minutes. Horizontal bar = 25 ms; vertical bar = 1 pA. (**P** and **R**) Mean *P_O_* versus voltage relationship. *n* = 5 cells. Solid lines are Boltzmann fits. (**S**) Graph of *P_O_* for single-channel data in **O** and **Q**. Mean ± SEM. *P* < 0.0001 by 1-way ANOVA; ****P < 0.0001 by Tukey’s multiple-comparison test.

Our model in which Rad mediates the adrenergic-induced increase in cardiomyocyte Ca_V_1.2 currents depends on the Rad concentration being less than saturating of the sarcolemma-embedded Ca_V_α-β complex, and therefore inhibiting only a fraction of Ca_V_1.2 channels. This inhibited subpopulation comprises the Ca_V_1.2 channels available to augment current with adrenergic stimulation (2). To study regulation under closer to native conditions, the stoichiometry of the components must be controlled. Indeed, our initial studies identifying Rad as the critical mediator for adrenergic regulation of Ca_V_1.2 were significantly advanced by our careful titration of Rad in our HEK293 cell experiments (6). Here, we turned to heterologous expression in *Xenopus* oocytes, in which protein expression can be controlled more easily (18). Expression of WT Rad in oocytes markedly reduced the current amplitude of heterologously expressed Ca_V_1.2 (Figure 7, A and B, and Supplemental Table 1) and produced a depolarizing shift of approximately 5 mV of *V_50_* for activation (Figure 7C). In oocytes expressing C-6SD Rad, basal currents were significantly increased compared with WT Rad (Figure 7, A and B) and the *V_50_* shifted back to that of Rad-free channels (Figure 7C).

We assessed the effects of changes in charge of the Rad CTD on intact Ca_V_1.2 open probability (*P_O_*). We used low-noise single-channel recordings of HEK293 cells transfected with WT or mutant Rad, α_1C_ and β_2B_. Exemplar records show that Rad-bound channels exhibit sparse channel openings (Figure 7E) compared with channels without Rad, which undergo high-activity flickery openings (Figure 7D). Ensemble average *P_O_*-voltage relationships from individual patches show a strong reduction in maximal *P_O_* of Rad-bound channels compared with Rad-less channels (Figure 7F). The addition Rad with just 2 Asp residues in the CTD of Rad caused strong reduction in *P_O_*, similar to the effect of WT Rad (Figure 7, G and H). The addition of 4 or 6 Asp residues in the CTD of Rad blunted the inhibitory effects of Rad (Figure 7, I–L). Taken together, our results show that the addition of 4 to 6 negative charges to the CTD of Rad mimics the stimulatory effects of β-adrenergic agonists on Ca_V_1.2 function.

The electrophysiological effects of adrenergic agonists on Ca^2+^ channels require not only phosphorylation of the Rad CTD but also the concomitant dissociation of Rad from the plasma membrane, as shown by the following results. Firstly, the addition of CAAX to the Rad C-terminus prevented the increase in single-channel *P_O_* otherwise imparted by introducing 6 negatively charged Asp residues (Figure 7, M and N). Secondly, the addition of CAAX to the Rad CTD prevented adrenergic regulation of Ca_V_1.2; in HEK293 cells heterologously expressing WT Rad and Ca_V_1.2 α_1C_ and β_2B_ subunits, forskolin plus calyculin increased the *P_O_* of Ca_V_1.2 channels (Figure 7, O, P and S), but not in cells expressing Rad with C-terminal CAAX (Figure 7, Q–S). Thus, phosphorylation of the C-terminus of Rad is insufficient to cause changes in Rad-β interaction or increased Ca^2+^ current without a concomitant dissociation of Rad from the membrane.

## Discussion

**Figure 8:**
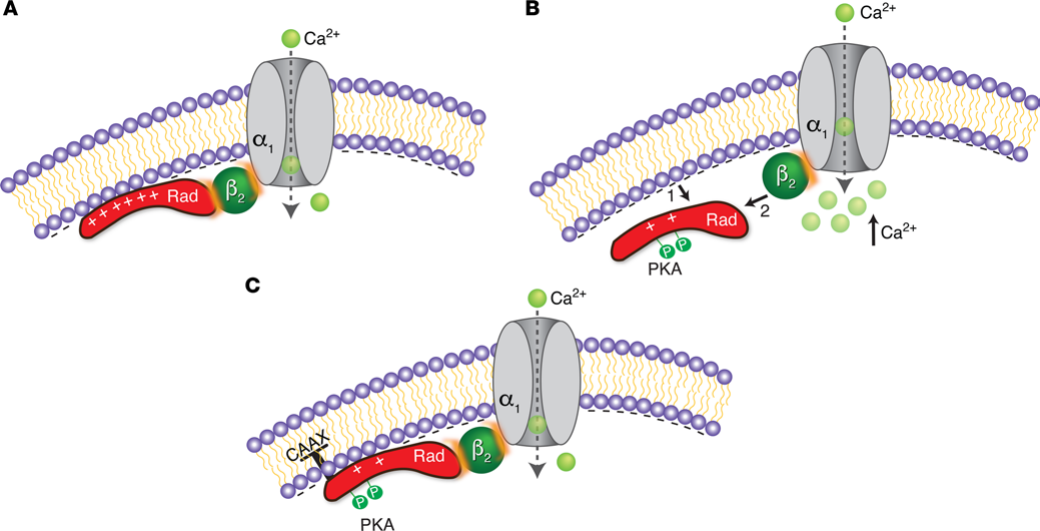
Schematics of role of membrane affinity and interaction of Rad and Ca_V_β in β-adrenergic regulation of Ca_V_1.2. (**A**) Ca_V_β is close to the membrane because it is bound to Ca_V_α, a membrane-embedded channel protein. In the basal state, the binding of Rad and Ca_V_β (and consequent channel inhibition) is promoted by the association of positively charged Rad with the negatively charged plasma membrane. Since the intrinsic affinity of Rad for β is low, the binding of Rad to β depends on the compensating, high local concentration of Rad at the membrane. Rad binding to β leads to inhibition of Ca^2+^ channels. (**B**) Adrenergic stimulation causes PKA activation and phosphorylation of the C-terminus of Rad, which decreases the net positive charge of the C-terminus of Rad. This change in charge disrupts Rad’s attachment to the membrane (arrow 1), thereby diluting it and favoring its dissociation from Ca_V_β (arrow 2). Rad-less Ca^2+^ channels have increased open probability compared with Rad-bound channels. (**C**) Rad is constitutively attached to the membrane via appending the CAAX motif to its C-terminus. Despite the change in charge induced by PKA phosphorylation of Rad, the CAAX motif prevents Rad’s detachment from the membrane, and thus the binding of Rad to Ca_V_β is preserved and the Ca^2+^ channel remains inhibited.

Rad acts through Ca_V_β to inhibit Ca_V_ channels (Figure 8A). Mutations in the sites of binding between Rad and Ca_V_β that eliminate the binding also eliminate Rad’s ability to inhibit Ca_V_ channels (37). The affinity of Rad for Ca_V_β is low (47); however, an effective interaction between Rad and Ca_V_β requires a high local concentration of Rad, achieved by attaching Rad to the plasma membrane where Ca_V_β is located through its interaction with the membrane-embedded α_1C_ pore-forming subunit (Figure 8A). β-Adrenergic control of the inhibition of Ca_V_ by Rad exploits Rad’s reversible attachment to the sarcolemma. PKA phosphorylation of Rad at 2 Ser residues in its CTD disrupts Rad’s attachment to the membrane, thereby diluting its local concentration and favoring its dissociation from Ca_V_β (Figure 8B). Asp residue substitutions of the 2 Ser in the CTD of Rad is insufficient to mimic the effects of phosphorylation because substitution with these Asp residues cause a change in charge of –2 compared with a change in charge of –4 imparted by 2 phosphates. If Rad is constitutively attached to the membrane via appending the CAAX motif to its C-terminus, PKA phosphorylation of Rad or Asp substitutions in the CTD fail to disrupt Rad’s attachment to the membrane and the binding of Rad to Ca_V_β is preserved (Figure 8C).

Our results explain how a substoichiometric amount of Rad inhibits a subpopulation of Ca_V_1.2 channels that serve as the reserve pool to increase Ca^2+^ currents in cardiomyocytes in response to sympathetic stimulation. Specifically, our findings here indicate that the binding of Rad and Ca_V_β (and consequent channel inhibition) is promoted by the association of positively charged Rad with the negatively charged plasma membrane (Figure 8A). It is, however, the charge of the Rad CTD, not the overall charge of Rad, that governs its association with the membrane. Lowering the positive charge of the Rad CTD by PKA phosphorylation of Ser^272^ and Ser^300^, as occurs (a) in vivo following adrenergic simulation, (b) in vitro by addition of PKA or forskolin and calyculin, or (c) by mutagenic substitution of CTD Ser residues by Asp weakens Rad binding to the membrane and to β. Adding negative charges to the NTD of Rad has a minor effect on the association of Rad with the membrane and with β (see Figure 4G for charge changes). As seen with other members of the RGK family (33), it is likely that an array of Arg and Lys residues interspersed with hydrophobic residues in the Rad CTD interacts with the negatively charged cytoplasmic leaflet of the cell surface bilayer, likely specifically with patches of phosphoinositides. The likely intrinsically disordered state of the Rad CTD (Figure 4A) may support its role of binding to laterally mobile phosphoinositides in the plasmalemma (48). This is in contrast to the role of the structured middle of Rad in binding to β. Given that β in its complex with Ca_V_α is necessarily oriented, so must the orientation of Rad imposed by the binding of its CTD to the membrane impose on Rad the appropriate orientation for binding to β. There is also the possibility that the binding of the Rad CTD to the membrane, acting allosterically, alters the structure of its binding site for β, increasing its affinity.

Partitioning of proteins to the cell membrane surface can increase their local concentration relative to their cytoplasmic concentration by orders of magnitude, depending on their net positive charge and the surface electrostatic potential (32, 49, 50). In the case of Rad, the number of Arg and Lys exceed the number of Asp and Glu by 8; a rough estimate of its overall charge is 8.8 (Figure 4G). If the electrostatic potential of the membrane surface were –35 mV (40), then [Rad]_membrane_/[Rad]_cytoplasm_ = exp(–*z* × *F* × *V*/[*R* × *T*]) = 6 × 10^4^, where *z* is algebraic charge, *F* is the Faraday constant (96,487 coulombs/mole), *V* is electrostatic potential (in volts), *R* is the gas constant (8.314 Joules/[degrees Kelvin × mole]), and *T* is degrees Kelvin. Phosphorylation of just 2 serine residues in the CTD would reduce the net charge on Rad from approximately 8 to 4 and would result in [Rad]_membrane_/[Rad]_cytoplasm_ = 250, a 250-fold decrease in the ratio. Just the C-terminal last 57 residues of Rad, the CTD, has an excess of Arg and Lys over Asp and Glu of 14. At V_mem_ = –35 mV, [CTD]_membrane_/[CTD]_cytoplasm_ = 2 × 10^8^. Adding 2 phosphates to the CTD would reduce its charge to 10 and its concentration next to the membrane relative to the bulk cytoplasm also by 250-fold. These simple calculations show that Rad could be highly concentrated at the cytoplasmic surface of the plasmalemma, with its CTD closest to the membrane, and that lowering the positive charge of the CTD could lower its local concentration by 2 orders of magnitude. These rough calculations show that the electrostatic effects could be large.

Another consequence of the electrostatic potential of the cytoplasmic surface of the plasmalemma is the partition of protons. By the same calculation as above, at –35 mV, the pH at the membrane surface would be 6.6, assuming that the pH in the bulk cytoplasm were 7.2. Thus, the charge on proteins at the surface would increase because of the charging of histidyl residues, whereas the charges on Lys, Arg, Glu, and Asp would not be affected appreciably (Figure 4G).

Over what range would the surface potential have these effects? Taking 150 mM 1:1 salt to represent the ionic composition of the cytoplasm, the Debye length would be 0.785 nm (51). If V(0) = –35 mV at the membrane surface, then V(0.5 nm) = –18.5 mV, V(1 nm) = –9.8 mV, and V(2 nm) = –2.7 mV (eq. 5 in ref. 51). Thus, the juxtamembrane phase with highly concentrated Rad would be very thin. It is likely, moreover, based on the importance of CTD hydrophobic residues (33) that Rad in this region is not diffusing in 3 dimensions but rather is bound to the bilayer and diffusing with phosphoinositides in 2 dimensions. It is with Venus-Rad concentrated in this array that Cerulean-β_2B_ would interact and bind.

The approximate charge of β_2B_ at pH 7.2 (based on the pKa of residues in an unfolded polypeptide) is 5.6 and at pH 6.6, 14.4 (Figure 4G). This large difference is due to the charging of the 28 His residues in β_2B_. Again, note that these charges are for the residues in an unfolded polypeptide, but nonetheless, β_2B_ will bear a large positive charge. In cardiomyocytes, in the presence of Ca_V_α, β_2B_ is concentrated at the sarcolemma (Figure 1C). We do not know, however, the extent to which β_2B_ heterologously expressed in the absence of Ca_V_α is concentrated at the plasmalemmal surface. In contrast to the β_2A_ isoform, which is concentrated at the plasmalemma, β_2B_ was disperse (21). Even if β_2B_ is dispersed when expressed in HEK293 cells, its binding to Rad highly concentrated at the plasmalemmal surface would be highly enhanced compared with such binding in the cytoplasm. The dissociation constant of the Rad-β_2B_ complex is likely to be high, similar to the weak interaction (*K_d_* = 156 μM) between Rem2, a Rad-related small GTPase, and Ca_V_β_4_ subunits, as determined by isothermal calorimetry in a cell-free environment (47). Yet, Rem2 effectively inhibits α_1A_- and Ca_V_β_4_-containing P/Q Ca^2+^ channels (47). Significant binding would occur only when at least one of the partners is at a high concentration. Our measure of binding (Figures 2 and 4), FRET from Cerulean-β to Venus-Rad, is also more likely if a 2-dimensional array of Venus-Rad is in the close vicinity of bound Cerulean-β.

If phosphorylation and substitutions of Asp residues in the Rad CTD, which dissociate the Venus-Rad–Cerulean-β_2B_ complex, were not acting through dissociation of Venus-Rad from the membrane, then they would need to be acting to weaken the molecular interaction of Rad and β. However, these same modifications of Venus-Rad-CAAX, anchored in the membrane, do not affect its binding to Cerulean-β_2B_. It is highly likely then that the modifications decreasing the charge of the Rad CTD decrease FRET from Cerulean-β_2B_ to Venus-Rad, our measure of their association, by releasing Venus-Rad from the plasmalemma. In another example of kinase-regulated membrane association, Ste5, a MAPK cascade scaffold protein in yeast, regulates yeast differentiation via a phosphoregulated membrane-binding motif (52). In that case, as in ours, reproducing the effect of phosphorylation required the substitution of 2 Asp residues for each removed Ser or Thr target for phosphorylation (52).

Most members of the Ras superfamily of small GTP-binding proteins have CAAX motifs at their C-terminus, resulting in their strong association with the plasma membrane (53). The absence of the CAAX motif in the CTD of Rad, and potentially other RGK GTPases, is a prerequisite for phosphoregulation of membrane localization. Our finding that the combination of forskolin and calyculin does not increase the *P_O_* of Ca^2+^ channels when Rad is strongly tethered to the membrane by the prenylated CAAX motif confirms that phosphorylation of neither α_1C_ nor β_2B_ subunits is necessary or sufficient for adrenergic regulation of Ca_V_1.2 channels. This confirms our prior studies showing that the adrenergic regulation of heterologously expressed Ca_V_1.2 channels does not require phosphorylation of any Ser or Thr residue within these subunits (6, 7, 9, 10, 17). In summary, changing the electrostatic charge of the Rad CTD by inserting negatively charged Asp residues is fully sufficient to activate Ca^2+^ channels, independent of signaling pathways initiated by adrenergic agonists. Tuning the association of Rad with the cardiomyocyte sarcolemma could be a therapeutic approach to enhance or attenuate Ca^2+^ influx and adrenergic signaling in the heart.

## Methods

Additional experimental details can be found in the Supplemental Methods.

### Sex as a biological variable.

We used both male and female mice at 6 weeks to 5 months of age.

### Generation of mice.

Animals were maintained under a standard 12-hour light/12-hour dark cycle and had access to standard chow and water ad libitum. The investigators were blinded to group allocation during data acquisition and analysis.

The transgenic β_2_, β_3_, and β_4_ mutant mouse constructs were created by ligating in-frame a 3× FLAG epitope to human *CACNB2b* cDNA (accession AAG01473), rat *Cacnb3* (NM_012828), or *Cacnb4* (NM_001105733). All FLAG-β cDNAs were ligated into the pJG/α-myosin heavy chain (MHC) plasmid (a gift from Jeffrey Robbins, Addgene plasmid 55594) (54). The purified transgene fragments were injected into pronuclei of fertilized mouse eggs, which were then implanted into pseudopregnant females by the Genetically Modified Mouse Models Shared Resource at Columbia University. The transgenic mice, created in a B6CBA/F2 hybrid background, were bred with WT C57BL/6N.

The N-2SA Rad–knockin mouse line with Ala substitutions for the 2 N-terminal Ser residues and the *Cacnb2*-null mouse were created using CRISPR/Cas9 gene editing. Validation of the single guide RNA (sgRNA) and single-stranded oligodeoxynucleotide (ssODN) was performed in the Genome Engineering and iPSC Center (GEic) at Washington University. The sgRNA for β_2_ knockout was ATAGGACGGCTGGTTAAAGANGG. The sgRNAs and ssODNs for creation of the N-2SA Rad mice are shown in Supplemental Figure 3. Zygotes isolated from C57BL/6N mice were electroporated with the sgRNA and ssODN at Mount Sinai School of Medicine Mouse Genetics and Gene Targeting Core. Identification of potential founders and germ-line transmission after crossing with WT C57BL/6N mice was performed by deep sequencing of genomic DNA from tail biopsies. Heterozygous N-2A Rad offspring mice were crossed to obtain homozygotes.

### Clone construction and cell culture.

All Rad (accession XM_006531206) constructs were generated by gene synthesis (Gene Universal) or site-directed mutagenesis. All cDNA clones were authenticated by sequencing. HEK293T cells (ATCC, CRL-3216) were cultured in DMEM with 10% FBS and 1% Pen/Strep. We transfected rabbit α_1C_ (accession X15539), human β_2B_ (NM-201590.3), and WT and mutant Rad using Lipofectamine 2000 (Thermo Fisher Scientific). The media were changed 4–6 hours after transfection.

### Whole-cell patch clamp electrophysiology studies in cardiomyocytes.

Mouse ventricular myocytes were isolated by enzymatic digestion using a Langendorff perfusion apparatus, and cellular electrophysiology studies were performed as described previously (2, 7, 9, 10, 55, 56). Cells without a stable baseline (possibly due to rundown or run-up) were not studied. Capacitance transients and series resistance were compensated (>85%). Voltage was corrected for liquid junction potential (–10 mV) during analysis. Leak currents were subtracted by a P/3 protocol. The conductance was normalized to cell size. After the isolated cardiomyocytes were dialyzed and adequately buffered with 10 mM BAPTA in the internal solution, cells were locally superfused with 140 mM tetraethylammonium chloride (TEA-Cl), 1.8 mM CaCl_2_ (or 0.5 mM BaCl_2_ for ramp protocol), 1 mM MgCl_2_, 5 mM glucose, and 10 mM HEPES, adjusted to pH 7.4 with CsOH. To measure peak currents, we held the cell membrane potential at –60 mV and stepped it to +50 mV for 150 ms in 10-mV increments every 10 seconds. In many experiments, we used a ramp protocol with a 200-ms voltage ramp from –60 mV to +60 mV (0.6 V/s) applied every 3 seconds to monitor the I-V relationship. After establishing stable records (usually after 2–3 minutes), 10 to 15 traces were recorded for control. Thereafter, isoproterenol, forskolin, or isoproterenol plus calyculin A were superfused. After the response stabilized, typically within 2 to 3 minutes for isoproterenol and 3 to 6 minutes for forskolin, 10 to 15 additional traces were recorded. When no response was observed, we continued the experiments for 4–6 minutes. The analysis of the ramp protocol experiments was previously described (6).

Forskolin (Santa Cruz Biotechnology, sc-3562) was used at a concentration of 10 μM or 50 μM made from a 10 mM stock dissolved in DMSO. Isoproterenol (Sigma-Aldrich, I5627) was applied at a concentration of 200 nM. EDTA (50 μM) was added to prevent rapid degradation of isoproterenol (57).

### Whole-cell patch clamp studies of Ca_V_1.2- and Rad-transfected HEK293 cells.

Electrophysiological recordings were carried out at room temperature 24–48 hours after transfection. After establishing a seal and achieving whole-cell configuration, external solutions were changed by the fast local perfusion method. Cells were kept at a holding potential of –50 mV. We used a ramp protocol to monitor the I-V relationship. Tail current amplitude was measured upon return to –10 mV.

### Single-channel recordings.

Single-channel recordings were performed at room temperature as described previously (6). The pipette solution contained 140 mM tetraethylammonium methanesulfonate, 10 mM HEPES, and 40 mM BaCl_2_ at 300 mOsm/L adjusted with tetraethylammonium methanesulfonate, and pH 7.4 adjusted with tetraethylammonium hydroxide. To zero the membrane potential, we placed the cells in a bath containing 132 mM potassium glutamate, 5 mM KCl, 5 mM NaCl, 3 mM MgCl_2_, 2 mM ethylene glycol-bis(β-aminoethyl ether)-*N*,*N*,*N*′,*N*′-tetraacetic acid (EGTA), 10 mM glucose, and 20 mM HEPES at 300 mOsm/L adjusted with glucose and pH 7.4 adjusted with KOH. Single-channel currents were elicited by 200-ms voltage ramps from –80 to +70 mV (portions between –60 and +40 mV displayed and analyzed) and were filtered at 2 kHz using a low-pass Bessel filter. In some experiments, forskolin and calyculin were added to the bath at a final concentration of 10 mM and 100 nM, respectively. Recordings were then obtained within 10 minutes of initial treatment. For each patch, the ensemble average currents were obtained by averaging 80 to 120 sweeps with a repetition interval of 5 to 10 seconds. BayK 8644 (Tocris, 1544) was then added to the bath at a final concentration of 9 μM to count the total number of channels in each patch, obtaining 50–120 additional sweeps. Recordings were obtained from cells from at least 2 different transfections. The average *P_O_* at +30 mV was calculated following idealization using a half-height detector.

### Whole-cell 2-electrode voltage clamp in Xenopus oocytes.

Frog maintenance and surgery and DNA and RNA preparation were done as described previously (18). Each oocyte was injected with 5 ng of RNAs for rabbit Ca_V_1.2 subunits α_1C_, β_2B_, and α_2_δ_1_ (GenBank: M21948) and 1 ng mouse Rad. Channel currents from oocytes were recorded using a 2-electrode voltage clamp. Ca^2+^ channel currents were recorded in 40 mM Ba^2+^ solution: 40 mM Ba(OH)_2_, 50 mM NaOH, 2 mM KOH, and 5 mM HEPES, titrated to pH 7.5 with methanesulfonic acid. At the start of the recording, depolarizing pulses from –80 mV to +20 mV were applied at 10-second intervals until the amplitude of I_Ba_ has stabilized and the current was constant for at least 2 minutes before applying the I-V protocol. Pulses (20 ms) were applied from the holding potential –80 mV to voltages from –70 to +70 mV, with 10-mV steps and 10-second intervals between sweeps. Then, the currents were blocked with 200 μM Cd^2+^ and the net I_Ba_ at each voltage was obtained by subtracting the currents recorded in the presence of Cd^2+^. For each cell, the I-V curve data were fitted using Boltzmann’s equation.

### Fractional shortening of isolated cardiomyocytes.

Freshly isolated myocytes were superfused with a Tyrode’s solution containing 1.2 mM CaCl_2_. Myocytes were field stimulated at 1 Hz. Percentage contraction of sarcomere length was measured using the SarcLen module of Ionoptix and calculated as the difference of shortest sarcomere length during a contraction subtracted from the relaxed sarcomere length, divided by the relaxed sarcomere length, all averaged over at least 8 contractions. Forskolin was used at concentration of 10 μM.

### Immunoprecipitation and immunoblots.

Cardiomyocytes were lysed with a hand-held tip homogenizer in a 1% (v/v) Triton X-100 buffer containing 50 mM Tris-HCl (pH 7.4), 150 mM NaCl, 10 mM EDTA, 10 mM EGTA, cOmplete Mini Protease inhibitor tablet (Roche), and PhosSTOP (Roche). Proteins were size-separated by SDS-PAGE, transferred to nitrocellulose membranes, and probed with either a custom-made rabbit polyclonal anti–α_1C_ subunit antibody (Yenzym; 1:1,000 dilution) (58, 59), a custom-made polyclonal anti–β subunit antibody (7), a custom-made polyclonal anti-Rad antibody (epitope: GSRGAGRERDRRRG, 1:1,000 dilution; Yenzym), anti–β-actin antibody (Santa Cruz Biotechnology, sc-47778; 1:1,000 dilution), and anti-FLAG antibody (Sigma-Aldrich, F7425; 1:1,000 dilution). Signal detection was performed with horseradish peroxidase–conjugated secondary antibodies and enhanced chemiluminescence using an Azure Biosystem 600 imager.

### Flow cytometric FRET 2-hybrid assay.

HEK293T cells (ATCC CRL-3216) were cultured in 12-well plates and transfected with Lipofectamine 2000 (Thermo Fisher Scientific, 11668019). Cerulean–WT β_2B_ and Venus-tagged WT and mutant Rad cDNA pairs (1 μg) were mixed in serum-free DMEM. FRET experiments were performed 1 day after transfection. The protein synthesis inhibitor cycloheximide (100 μM) was added to cells 2 hours before experimentation. For FRET measurements, we used an LSR II (BD Biosciences) flow cytometer, equipped with 405 nm, 488 nm, and 633 nm lasers for excitation and 18 different emission channels. Analysis was performed as described previously (6). The FRET analysis software is accessible on github at https://github.com/manubenjohny/FACS_FRET

For analysis of membrane localization using FRET, we cotransfected cDNA encoding WT or mutant Rad with membrane-localized Cerulean-CAAX. Subsequently, we measured FRET efficiencies from individual cells. Here, FRET between Cerulean-CAAX and Venus-tagged Rad results from random collision of the acceptor with the donor and is dependent on the local concentration of the acceptor, which may vary depending on whether the acceptor is localized to the membrane or cytosol. Analysis suggests that the linear slope when FRET efficiency is plotted against fluorescence intensity of the acceptor (*S*_A,direct_) is proportional to the fraction of acceptor localized to the membrane (*f_mem_*) (see Supplemental Methods). We estimated the linear slope by first binning *E_D_-S_A,direct_* and then calculating a linear slope using least-squares fit.

### Immunofluorescence of isolated cardiomyocytes.

Isolated cardiomyocytes were fixed for 15 minutes in 4% paraformaldehyde, washed with glycine/PBS twice, treated with 0.1% Triton X-100 (v/v) in PBS (PBST) for 5 minutes, and blocked with 3% bovine serum albumin (BSA; w/v) in PBS for 1 hour. Indirect immunofluorescence was performed using either rabbit anti-FLAG antibody (1:200) or rabbit anti-Ca_V_β_2_ antibody (1:200) (7), followed by Alexa Fluor 594–labeled goat anti-mouse antibody (1:200; Thermo Fisher Scientific, A11032).

### Membrane fluorescence analysis from confocal imaging.

For quantification of membrane fluorescence from confocal images of Venus-tagged Rad and variants, we used a MATLAB 2020 script (MathWorks) as summarized here. First, to segment cells automatically we created a binary image using adaptive thresholding with the imbinarize function in the Image Processing toolbox in MATLAB. Spatial filtering was used to ensure the entire cell was captured. Individual cells were segmented by separating distinct regions where pixels are 8-connected in the image using the bwlabel function. For each single cell that was segmented, we calculated the average intensity in each concentric ring identified using morphological operators, starting with the outermost ring until the centroid is reached. This iterative procedure resulted in a radially averaged fluorescence intensity profile from the centroid to the periphery of the cell. Relative membrane fluorescence was then calculated as the ratio of maximal intensity at the periphery normalized to that at the centroid.

### Statistics.

Results are presented as mean ± SEM. For multiple group comparisons, 1-way ANOVA followed by multiple comparison testing was performed. For comparisons between 2 groups, an unpaired, 2-tailed *t* test was used. Statistical analyses were performed using Prism 8 (GraphPad). Differences were considered statistically significant at *P* values of less than 0.05.

### Study approval.

This study conformed to the NIH *Guide for the Care and Use of Laboratory Animals* (National Academies Press, 2011) and protocols approved by the Institutional Animal Care and Use Committee (IACUC) of Columbia University. *Xenopus* experiments were approved by the Tel Aviv University IACUC (permit 01-20-083).

### Data availability.

The data and study materials will be made available to other researchers for purposes of reproducing the results or replicating the procedure. Supporting data for all values underlying the data presented in the graphs are provided in the Supporting Data Values file.

## Author contributions

SOM and MBJ conceived the study. SOM, MBJ, AP, ANK, SIZ, LY, JAH, SW, ND, GSP, and HMC determined the study methodology. AP, PJRM, BXC, LY, ANK, SIZ, GL, VZ, MK, and SS carried out the investigation. SOM, MBJ, AK, and AP wrote the original draft of the manuscript. PJRM, ANK, SIZ, MSB, JAH, ND, GSP, and HMC reviewed and edited the manuscript. SOM, HMC, GSP, ND, and JAH acquired funding for the study. SOM, MBJ, ND, and JAH contributed resources to the study. The order of co–first authorship was assigned based on relative contribution to the manuscript.

## Supplementary Material

Supplemental data

Unedited blot and gel images

Supporting data values

## Version 1 01/16/2024

In-Press Preview

## Version 2 03/01/2024

Electronic publication
