# Supplementary material for: A membrane-associated phosphoswitch in Rad controls adrenergic regulation of cardiac calcium channels
Source: J Clin Invest. 2024 Jan 16;134(5):e176943. doi: 10.1172/JCI176943 (PMC10904049; doi:10.1172/JCI176943)
Supplement: Supplemental data [file jci-134-176943-s036.pdf]

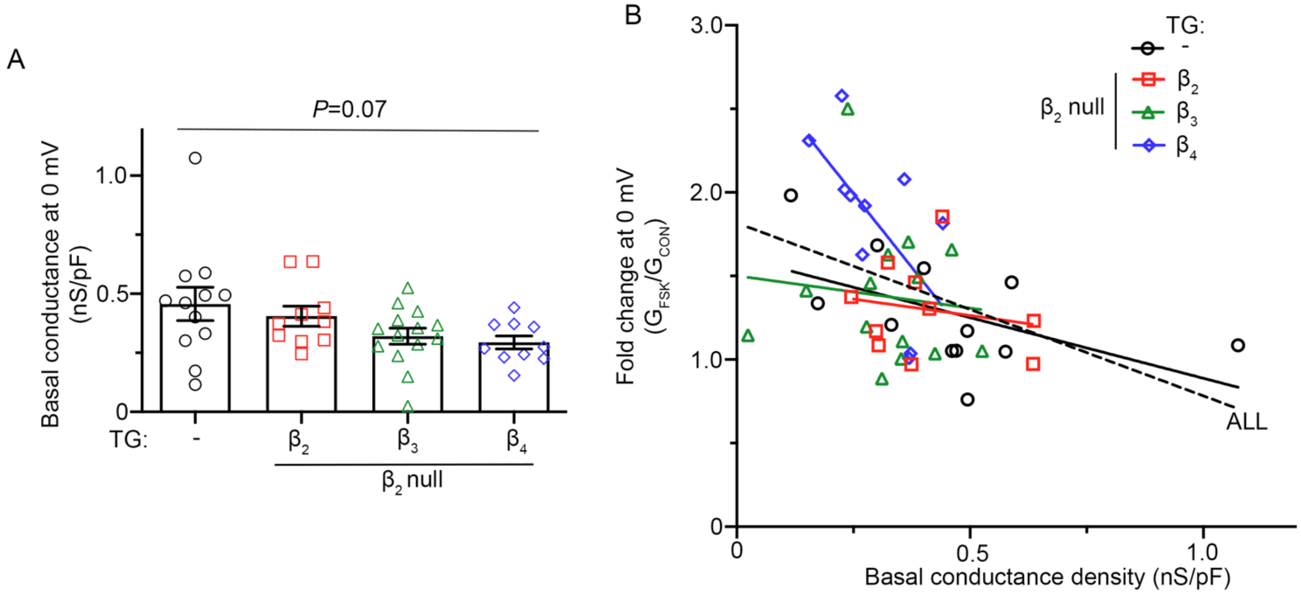


**Supplemental Figure 1. Electrophysiological properties of $Ca^{2+}$ channels with transgenic β subunits. (A)** Basal conductance density from non-transgenic WT mice, and transgenic mice expressing $β_2$, $β_3$ and $β_4$ in background of endogenous $β_2$-null cardiomyocytes. *P*= 0.07 by one-way ANOVA. **(B)** Forskolin (FSK)-induced fold increase in conductance stratified by basal conductance density. Lines were fitted by linear regression. The "ALL" line is the combination of all data points.

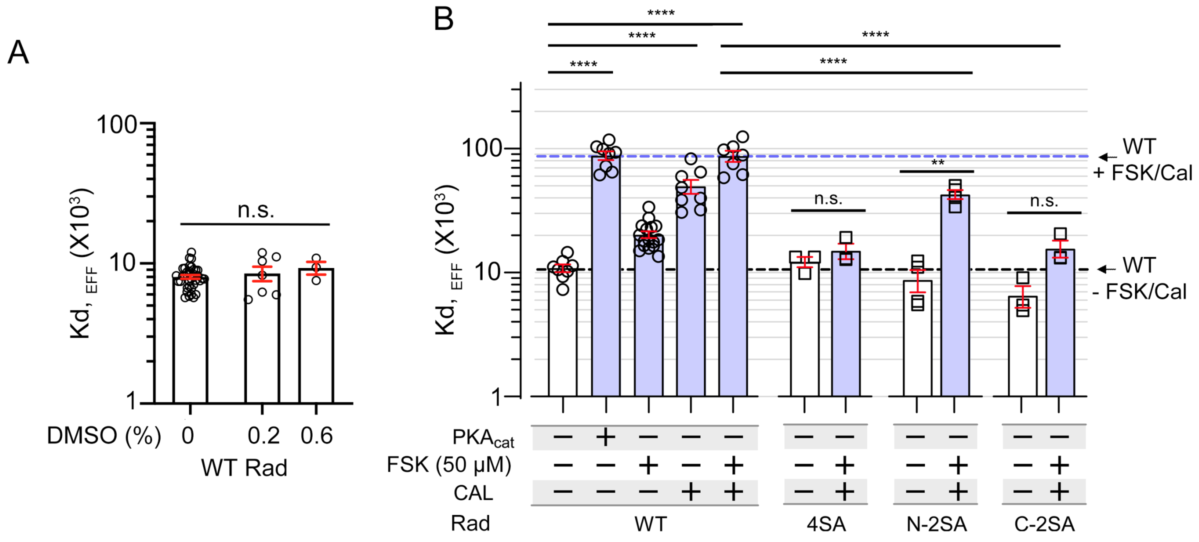


**Supplemental Figure 2. Adrenergic agonist induced disruption of β-Rad interaction. (A)** Graph summarizing mean $K_{d,EFF}$ for the binding of $\beta_{2B}$ and WT Rad in the absence and presence of 0.2% and 0.6% DMSO. For 10 μM forskolin and 100 nM calyculin experiments, the vehicle was 0.2% DMSO. For 50 μM forskolin and 100 nM calyculin experiments, the vehicle was 0.6% DMSO. *P*>0.05 by one-way ANOVA. **(B)** Graph summarizing mean $K_{d,EFF}$ for the binding of $\beta_{2B}$ and WT, 4SA Rad, N-2SA Rad and C-2SA Rad, in absence and presence of $PKA_{cat}$, 50 μM forskolin (FSK) and 100 nM calyculin A (Cal). Black dashed line is mean of WT Rad without FSK + Cal. Error bars represent SEM. *P*<0.0001 by one-way ANOVA, ***P* <0.01, *****P*<0.0001 by Sidak's multiple comparison test. N= 8,8,16,8,7,3,3,4,4,3,3 from left to right. First two columns same as Figure 2C.

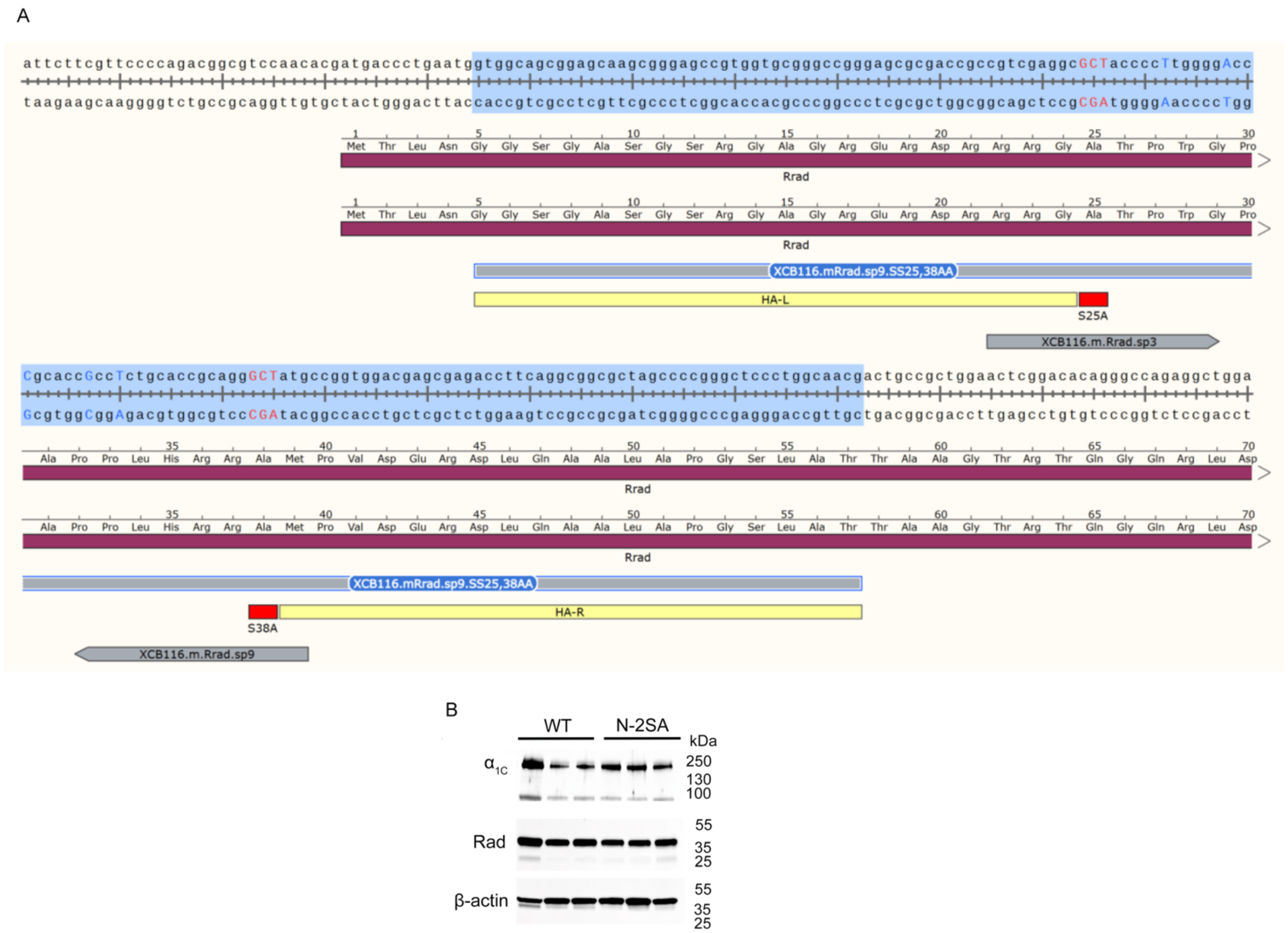


**Supplemental Figure 3. Creation of N-2SA knock-in mouse line. (A)** Schematic depicting approach for the creation of N-2SA knock-in mouse line. Guides and single-strand oligodeoxynucleotide as the donor template with Ala-substitutions for the two Ser residues were designed by Genome Engineering and iPSC Center (GEiC) at the Washington University in St. Louis. HA-L= homology arm-left, HA-R= homology arm-right. **(B)** Anti-$\alpha_{1C}$, anti-Rad and anti-β-actin immunoblots of protein homogenates from WT and N-2SA knock-in mice cardiomyocytes. N=3 mice for each genotype.

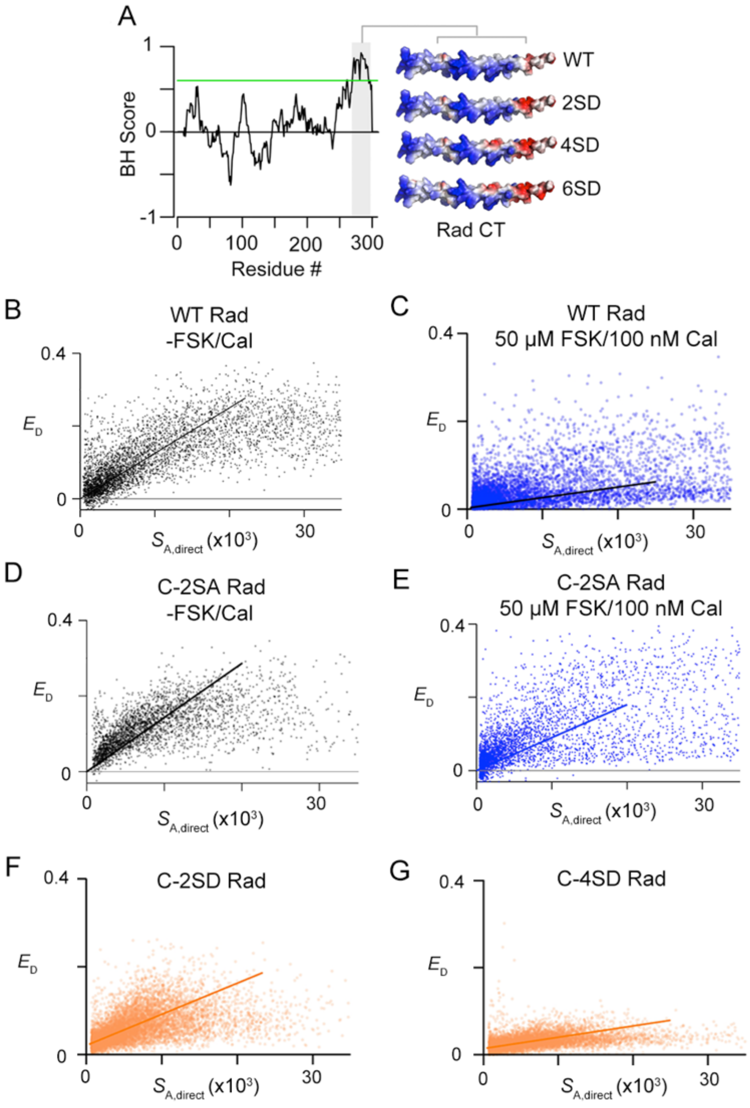


**Supplemental Figure 4. Change in membrane association of Rad induced by altering electrostatics in its C-terminus. (A)** Graph of basic hydrophobic (BH) score for the full sequence of Rad, created using a basic-hydrophobic scoring algorithm (36, 41). The x-axis is Rad residue numbers. The y-axis is BH score. BH motifs are defined by peaks with a BH signal > 0.6 (green line). On the right, a Pymol-generated model of C-terminus of Rad. Positive-charged amino acid residues are blue; negative charged amino acid residues are red. **(B-C)** $E_D$ is plotted against $S_{A,direct}$ of Ven-WT Rad, either untreated or treated with 50 μM forskolin + 100 nM calyculin A. **(D-E)** As in B-C, with C-2SA Rad. **(F-G)** $E_D$ is plotted against $S_{A,direct}$ of Ven-C-2SD Rad and Ven-C-4SD Rad in the absence of forskolin and calyculin A.

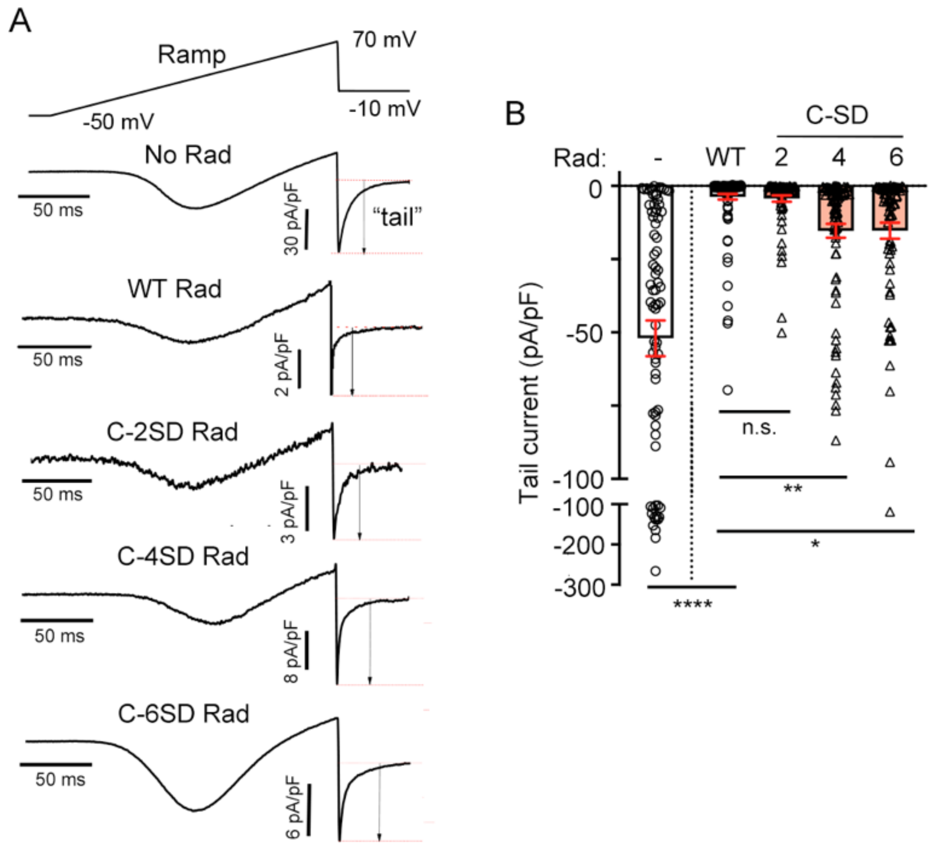


**Supplemental Figure 5. Insertion of negatively charged Asp residues in C-terminus of Rad reduces Rad-mediated inhibition of heterologously expressed $Ca^{2+}$ channels. (A)** $Ba^{2+}$ current elicited by voltage ramp every 6 seconds. Tail current is marked by arrows. **(B)** Graph of tail current from HEK293 cells heterologously expressed $\alpha_{1C}$ and $\beta_{2B}$, and no Rad, WT Rad or Rad with 2, 4, or 6 Asp-residues in C-terminus. Mean ± SEM. ****$P$<0.0001 by one-way ANOVA, *$P$<0.05, **$P$<0.01 compared to WT Rad by Dunnett's multiple comparison test.

| | $G_{max}$, µS | | $V_{rev}$, mV | | $V_{50}$, mV | | $K_a$, mV | |
|---|---|---|---|---|---|---|---|---|
| | mean | SEM | mean | SEM | mean | SEM | mean | SEM |
| $Ca_V1.2$ | 48.06 | 3.59 | 68.67 | 1.48 | 3.88 | 0.50 | 7.70 | 0.14 |
| $Ca_V1.2$ + Rad WT | 10.22 **** | 1.41 | 67.30 | 1.31 | 8.95 **** | 0.43 | 8.45 * | 0.23 |
| $Ca_V1.2$ + Rad C-6SD | 31.66 ns, p=0.13 | 2.65 | 66.62 ns, p=0.49 | 0.62 | 4.90 ns, p=0.54 | 0.89 | 7.20 ns, p=0.17 | 0.15 |

**Supplemental Table 1. Parameters of Boltzmann equation fit for I-V curves of full-length $Ca_V1.2$.** The table shows mean and SEM values of fits. N=10 oocytes in each group. The statistical analysis was done on raw data (i.e. the values of each parameter in individual oocytes). The asterisks indicate statistically significant differences between parameters in each group vs. the control group ($Ca_V1.2$ alone). * $P<0.05$; **** $P<0.0001$; ns, not significant. Data for all parameters except $G_{max}$ were normally distributed (Shapiro-Wilk test). For $G_{max}$, we performed Kruskal-Wallis test followed by Dunn's multiple comparison test. For the other parameters, one-way ANOVA was applied, followed by Tukey pairwise comparison test.

**Determining Membrane Localization with FRET**

To monitor changes in Rad localization, we used FRET measurements between a plasma membrane-localized Cerulean fluorophore (the donor) and Venus-tagged Rad (the acceptor). Fluorescence measurements were obtained with a BD Biosciences LSRII flow cytometer equipped with appropriate lasers to excite the donor and the acceptor and bandpass filters to separate the fluorescence emissions. Three fluorescence measurements were obtained from single cells: (1) donor fluorescence emission (excitation: 405 nm and emission/bandwidth: 450/50 nm) denoted as $S_D$, (2) acceptor fluorescence emission due to direct excitation (excitation: 488 nm and emission/bandwidth: 530/30 nm) denoted as $S_A(direct)$, and (3) acceptor fluorescence emission due to FRET (excitation 405 nm and emission/bandwidth: 525/50 nm) denoted as $S_A(FRET)$. Each cell is assumed to contain $N_D$ donor molecules and $N_A$ acceptor molecules. Donor and acceptor do not bind; proximity that supports FRET occurs by random motion. The donor is modified to be attached to the cytoplasmic surface of the plasmalemma and is thus confined to diffuse in two dimensions. A fraction of the acceptors ($f_{mem}$) is localized to the plasmalemma and the remaining fraction is assumed to diffuse in three dimensions. Significant FRET occurs only when the acceptor is close to the membrane-tethered donor.

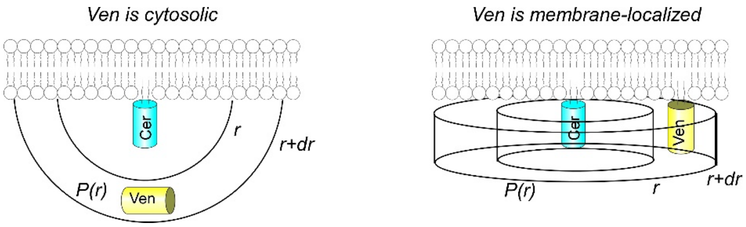


**Figure S1.** Schematics show a cerulean fluorophore localized to the membrane and relevant geometric arrangement for determining the probability *U*(r) of finding a Venus fluorophore within a distance *r* of Cerulean. Left, if Venus is cytosolically localized, it is assumed to diffuse in three dimensions. So, we consider the concentric hemi-spherical shells and the probability of a Venus being within this shell. Right, if Venus is membrane restricted then we assume two-dimensional diffusion. Thus, we consider thin cylindrical shells around Cerulean.

FRET efficiency ($E_D$) is obtained from the three fluorescence measurements as described previously (Erickson *et al* 2003). Briefly, the fluorescence outputs are:

$$\left.\begin{aligned} S_D &= N_D \cdot I_D \cdot G_D(\lambda_{ex,D}) \cdot (1 - E_D) \cdot F_D(\lambda_{em,D}) \\ S_{A,direct} &= N_A \cdot I_A \cdot G_A(\lambda_{ex,A}) \cdot F_A(\lambda_{em,A}) \\ S_{A,FRET} &= N_D \cdot I_D \cdot G_D(\lambda_{ex,D}) \cdot F_A(\lambda_{em,A}) \cdot E_D \end{aligned}\right\} \quad \text{Eq. 1}$$

*where* $N_D$ and $N_A$ are the numbers of molecules of donor and acceptor, $I_D$ and $I_A$ are the light intensities of the 405 nm and 488 nm lasers, respectively; $G_D(\lambda_{ex,D})$ and $G_A(\lambda_{ex,A})$ are instrument-specific constants that incorporate the optical properties of the instrument and the extinction coefficients of the donor and the acceptor, respectively; $F_D(\lambda_{em,D})$ and $F_A(\lambda_{em,A})$ are the outputs of the detector in response to the emissions of an excited donor and an excited acceptor.

$E_D$ is dependent on the distance (r) between the donor and the acceptor by the Förster relation:

$$E_D(r) = \frac{R_0^6}{r^6 + R_0^6} \quad \text{Eq. 2}$$

where $R_0$ is the Förster distance. $R_0$, in turn, depends on the spectral overlap of the fluorophores, the quantum yield of the donor, and the relative orientation of the fluorophores. This analysis assumes no specific binding of donor and acceptor; rather their distance and relative orientation are determined by random motion. Also, this relation assumes that at most one acceptor is near the donor. Thus, if $U(r)$ denotes the probability density and U(r)dr the probability that for a given donor, there is an acceptor within a distance between $r$ and $r + dr$, then the expected value of FRET efficiency is given by:

$$E_D = \int_0^\infty U(r) \cdot E_D(r) \cdot dr \quad \text{Eq. 3}$$

As acceptors can be localized to either the cytoplasm or restricted to the plasmalemma,

$$U(r) = U_{cyt}(r) + U_{mem}(r)$$

where $U_{cyt}(r)$ and $U_{mem}(r)$ are probability density functions for acceptors in the cytoplasm and the plasmalemma, respectively.

$$E_D = \int_0^{\infty} \left(U_{\mathrm{cyt}}(r) + U_{\mathrm{mem}}(r)\right) \cdot \mathrm{E_D}(r) \cdot dr \qquad \text{Eq. 4}$$

Here, for $r$ larger $R_0$, $E$ rapidly converges to 0. As such, the upper limit of the definite integral can be regarded as ∞.

Let $N_A$ be the total number of molecules of acceptors, and $f_{mem}$ is the fraction of acceptors associated with the membrane. $N_A$*(1-$f_{mem}$) is the total number of molecules in the cytoplasm of a cell with volume $V_{cell}$. The probability of finding an acceptor within a hemispherical shell of radius $r$ and thickness d$r$ centered on the donor is given by:

$$U_{\mathrm{cyt}}(r) \cdot dr = \frac{N_A \cdot (1 - f_{mem})}{V_{cell}} \cdot 2\pi r^2 \cdot dr \qquad \text{Eq. 5}$$

Similarly, if $N_A$* $f_{mem}$ acceptors are localized to the plasmalemma with surface area $S_{Cell}$, then the probability of finding an acceptor within the annulus of radius $r$ and width dr is:

$$U_{\mathrm{mem}}(r) \cdot dr = \frac{N_A \cdot f_{mem}}{S_{cell}} \cdot 2\pi r \cdot dr \qquad \text{Eq. 6}$$

We assume a low concentration of acceptors, such that there is only one acceptor near the donor. Thus, the measured FRET efficiency can be determined by substituting Eqs. 2, 5, and 6 into Eq. 4.

$$E_D = \int_0^{\infty} \left(\frac{N_A}{V_{cell}} \cdot 2\pi r^2 \cdot (1 - f_{mem}) + \frac{N_A}{S_{cell}} \cdot 2\pi r \cdot f_{mem}\right) \cdot \frac{R_0^6}{r^6 + R_0^6} \cdot dr$$

$$= \left[\frac{\frac{1}{3} \cdot \pi^2 \cdot R_0^3}{V_{cell}} + \left(\frac{\frac{2\sqrt{3}}{9 \cdot \pi^2} \cdot R_0{}^2}{S_{cell}} - \frac{\frac{1}{3} \cdot \pi^2 \cdot R_0^3}{V_{cell}}\right) \cdot (f_{mem})\right] \cdot N_A \qquad \text{Eq. 7}$$

If the cell is spherical with radius $a$, then $V_{cell} = \frac{4}{3}\pi a^3$ and $S_{cell} = 4\pi a^2$. Eq. 7 simplifies to:

$$E_D = \frac{\pi}{2} \cdot \left(\frac{R_0}{a}\right)^2 \left[\frac{1}{2}\left(\frac{R_0}{a}\right) + \left(\frac{1}{3\sqrt{3}} - \frac{R_0}{2a}\right) \cdot (f_{mem})\right] \cdot N_A \qquad \text{Eq. 8}$$

For Cerulean-Venus FRET pair, $R_0$ ~ 5.2 nm. The average radius of a HEK293 cell, $a$ ~ 6.5 µm. Thus, $R_0$ / $a$ < 0.001 For a sufficiently large $f_{mem}$, this relationship simplifies to:

$$E_D \sim \frac{\pi}{6\sqrt{3}} \cdot \left(\frac{R_0}{a}\right)^2 \cdot f_{mem} \cdot N_A \qquad \text{Eq. 9}$$

From Eq. 1, $N_A = S_{A,direct}/(I_A \cdot G_A(\lambda_{ex,A}) \cdot F_A(\lambda_{em,A}))$. Thus,

$$E_D \sim \left(\frac{\frac{\pi}{6\sqrt{3}} \cdot \left(\frac{R_0}{a}\right)^2}{I_A \cdot G_A(\lambda_{ex,A}) \cdot F_A(\lambda_{em,A})}\right) \cdot f_{mem} \cdot S_{A,direct} \qquad \text{Eq. 9}$$

Therefore, if $E_D$ is plotted as a function of $S_{A,direct}$, then the initial slope (i.e. low acceptor concentration) is directly proportional to the fraction of acceptors localized to the plasmalemma. At high acceptor concentrations, it is possible that multiple acceptors are present near the donor. As result, Eq. 2 would require additional terms to account for possible FRET transfer with multiple acceptors, ultimately leading to nonlinearities in the $E_D$-$S_{A,direct}$ relationship.

## References

Erickson, M.G., Alsheikhan, B.A., Peterson, B.Z., and Yue, D.T. (2001) Preassociation of calmodulin with voltage-gated Ca(2+) channels revealed by FRET in single living cells. Neuron 31:973-85.
