# Supplementary figures and images for: A membrane-associated phosphoswitch in Rad controls adrenergic regulation of cardiac calcium channels
Source: J Clin Invest. 2024 Jan 16;134(5):e176943. doi: 10.1172/JCI176943 (PMC10904049; doi:10.1172/JCI176943)

Uncropped gels  
Supplemental Figure 3A

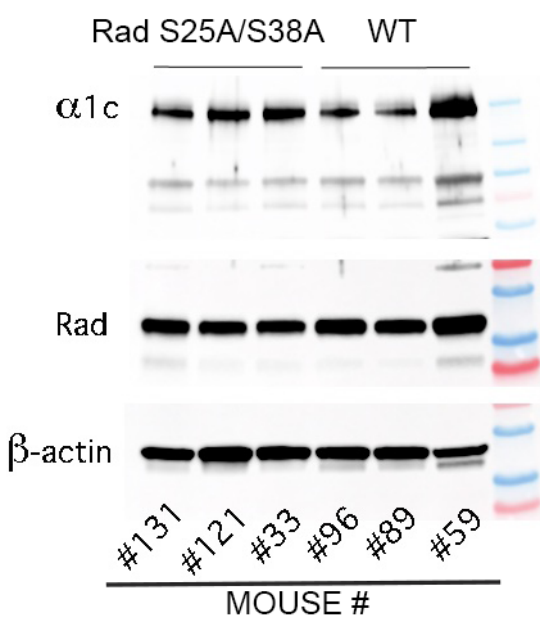

Supplement: Unedited blot and gel images [file jci-134-176943-s037.pdf]
